# A digital-health multidomain lifestyle management framework and its associations with cardiometabolic health: a real-world observational study

**DOI:** 10.1186/s12916-026-04830-y

**Published:** 2026-03-25

**Authors:** Han Xiao, Kexin Ding, Xiaoyi Li, Yujia Ma, Yan Liu, Jing Huang, Dafang Chen

**Affiliations:** 1 Department of Epidemiology and Biostatistics, School of Public Health, MRC Centre for Environment and Health, Imperial College London, London, UK; 2 Department of Epidemiology and Biostatistics, School of Public Health, Peking University, Beijing, China; 3 Key Laboratory of Epidemiology of Major Diseases (Peking University), Ministry of Education, Beijing, China; 4 Department of Non-Communicable Chronic Disease Control and Prevention, Beijing Center for Disease Prevention and Control, Beijing, China; 5 Department of Epidemiology and Health Statistics, School of Public Health, Capital Medical University, Beijing, China; 6 Yingdong Intelligent Technology (Shandong) Co., Ltd, Beijing, China; 7 Department of Occupational and Environmental Health Sciences, Peking University School of Public Health, Beijing, China; 8 Institute for Global Health and Development, Peking University, Beijing, China

**Keywords:** Digital health, Cardiometabolic health, Older adults, Health management

## Abstract

**Background:**

It remains unclear whether digital health-based multidomain management is associated with long-term cardiometabolic health control in real-world scenarios.

**Methods:**

We conducted a retrospective analysis of a real-world health management program involving 40,216 adults from 2018 to 2024 across 30 provinces in China. The program combined digital health platforms (including mobile health apps and smartwatches) with offline wellness centers to provide personalized lifestyle management. Participants' demographics, physiological (blood pressure, blood glucose, body composition, heart rate, grip strength, and cholesterol), psychological, and lifestyles (sleep and physical activity) data were longitudinally collected. Program engagement was measured by mobile health app usage frequency, device wear time, and wellness center participation. The population-level trajectory of cardiometabolic health changes was fitted over a 12-month follow-up using the linear mixed-effects models. Associations between participants’ engagement and cardiometabolic health (blood pressure, blood glucose, and body fat percentage) improvement were further examined.

**Results:**

The median (IQR) age of 40,216 participants was 71 (64–75). During the 5-year management period, participants contributed a total of 9,749,898 physical activity, 8,623,004 heart rate, 8,323,161 sleep, 2,925,903 body composition, 2,619,725 blood pressure, and 291,240 blood glucose data. Over the 5 years, participants used mobile health app to upload their health data for an average of 5.7 days per week, and wore smartwatches 5.8 days per week, with 95.5% wearing them for ≥ 8 h per valid day. Older age, female sex, and higher education level were associated with higher weekly frequency of mobile health app usage. Among participants with elevated blood pressure, elevated blood glucose, or obesity, those with high engagement were associated with greater reductions in systolic blood pressure (− 3.85 [-5.2 to -2.49] mmHg), blood glucose (− 1.20 [ -2.06 to -0.34] mmol/L), and body fat percentage (− 1.24% [-1.59% to -0.88%]). Explorative mediation analyses suggested that reductions in body fat percentage partially mediated these associations.

**Conclusions:**

Findings of this study suggest that a digital health-based multidomain program may support daily cardiometabolic health monitoring and management. Such frameworks can be feasible and scalable to help facilitate proactive prevention strategies.

**Supplementary Information:**

The online version contains supplementary material available at 10.1186/s12916-026-04830-y.

## Background

China is becoming a severely aging society. With an elderly population (aged over 60) expected to reach 402 million by 2040 [1], China faces significant challenges to public health and healthcare systems [2, 3]. Central to this challenge is the management of cardiometabolic diseases, which accounted for 49.8% of total mortality in the decade from 2009 to 2019 [4, 5]. The high prevalence of cardiometabolic diseases and their multi-morbidities is substantially attributed to unhealthy lifestyles, such as high-calorie diets and sedentary lifestyles. While these factors underscore the importance of continuous monitoring and early intervention in mitigating disease progression [6], the traditional care model struggles to meet the needs for long-term daily care. Addressing this requires a strategic shift toward increasing the availability and affordability of medicines and integrated care [7].

Digital health offers new approaches to support such transitions from traditional medical routines to remote, long-term daily care for older adults. The use of digital health technologies could support lifestyle interventions, monitor chronic disease indicators, and enhance doctor-patient communication, all of which are crucial for managing cardiometabolic diseases [4]. Recent studies have highlighted the clinical use of digital health technologies in blood pressure (BP) [8, 9], blood glucose (BG) [10, 11], and weight self-management [12]. While growing literature demonstrated the positive effects of digital health in providing personalized healthcare plans and improving patient adherence, translating these interventions into real-world settings remains a challenge. As such, real-world evidence (RWE) is crucial for evaluating the actual implementation of digital health interventions in everyday practice, which can further inform healthcare decisions [13].

In the past five years, numerous practices and innovations for digital health have emerged in China. Strategies such as "Internet + Healthcare" have been integrated into the "Healthy China 2030" planning framework, promoting the development of telemedicine and internet hospitals [14]. Nevertheless, empirical evidence regarding the large-scale implementation of digital health interventions within community-based aging populations remains insufficient. As such, YIDO is a multi-domain cardiometabolic health management program, characterized by the combination of several lifestyle interventions, that covers users from almost all regions of China. It is administered via integrated digital health technologies and community-based wellness centers, creating a closed-loop system that links multidimensional data collection, health risk assessment, personalized interventions, health education, and daily follow-up. Participation in the program has been associated with decreased BP and weight [15, 16].

The present study aims to introduce this digital-health multidomain lifestyle management framework for cardiometabolic health and report descriptive real-world data collected during a 5-year management period. Furthermore, we investigated the influencing factors of program engagement. We also evaluated whether program engagement was associated with improvements in cardiometabolic metrics, including BP, BG, and body fat percentage.

## Methods

### Study design and participants

**Fig. 1:**
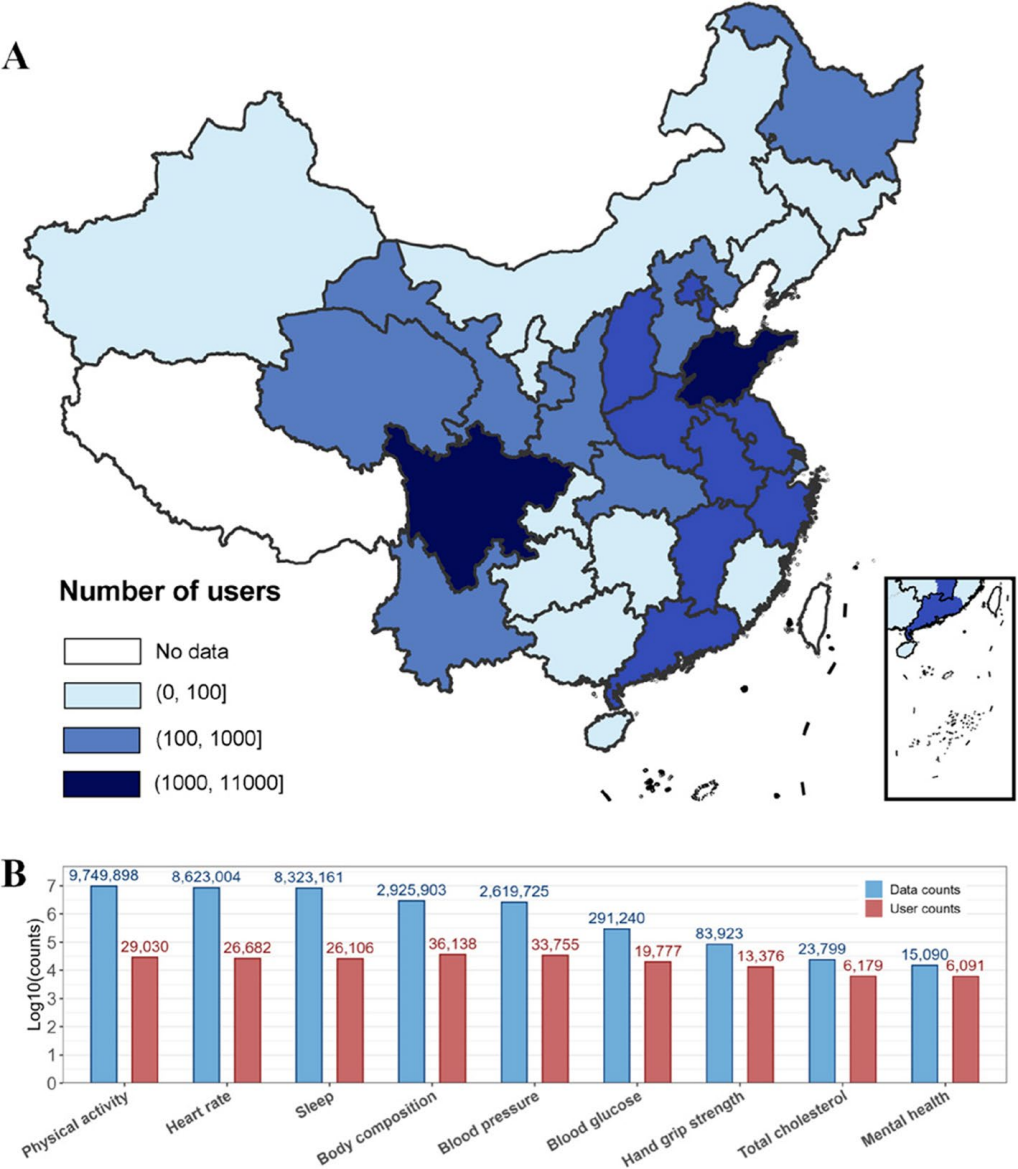
**A** The geographical distribution of participants in China by provinces. **B** The number of users and data counts in the nine main datasets of the program. “User count” is defined as the number of unique users with ≥ 1 valid record in the dataset, and “data count” is the total number of uploaded measurements in the dataset

This is an observational study of a real-world digital health management program. Participants joined this program to improve cardiometabolic health by targeting modifiable lifestyles. Participants were enrolled in this commercial program at any time starting from January 2018 in China (Fig. 1). Eligibility criteria included being 18 years or older, fluency in Chinese, owning a smartphone, possessing available disease history information, and regularly accessing the app to upload daily activity data throughout the management period. Participation also required costs to the program’s mobile health app and the use of home monitoring devices (e.g., smart body composition scale). Retrospective analyses were conducted using participants’ data collected in an anonymous and aggregated dataset without personal identifiers. Consent for participation in this study was obtained electronically when users created their accounts and accepted the treatment of personal data anonymously for research purposes. The study protocol and instruments were reviewed and approved by the Ethical Review Committee of Peking University in 2018 (IRB00001052-18039). The study protocol was registered in the Chinese Clinical Trial Registry (ChiCTR190023355).

### Components of the multidomain intervention framework

#### Digital health Platform

**Fig. 2:**
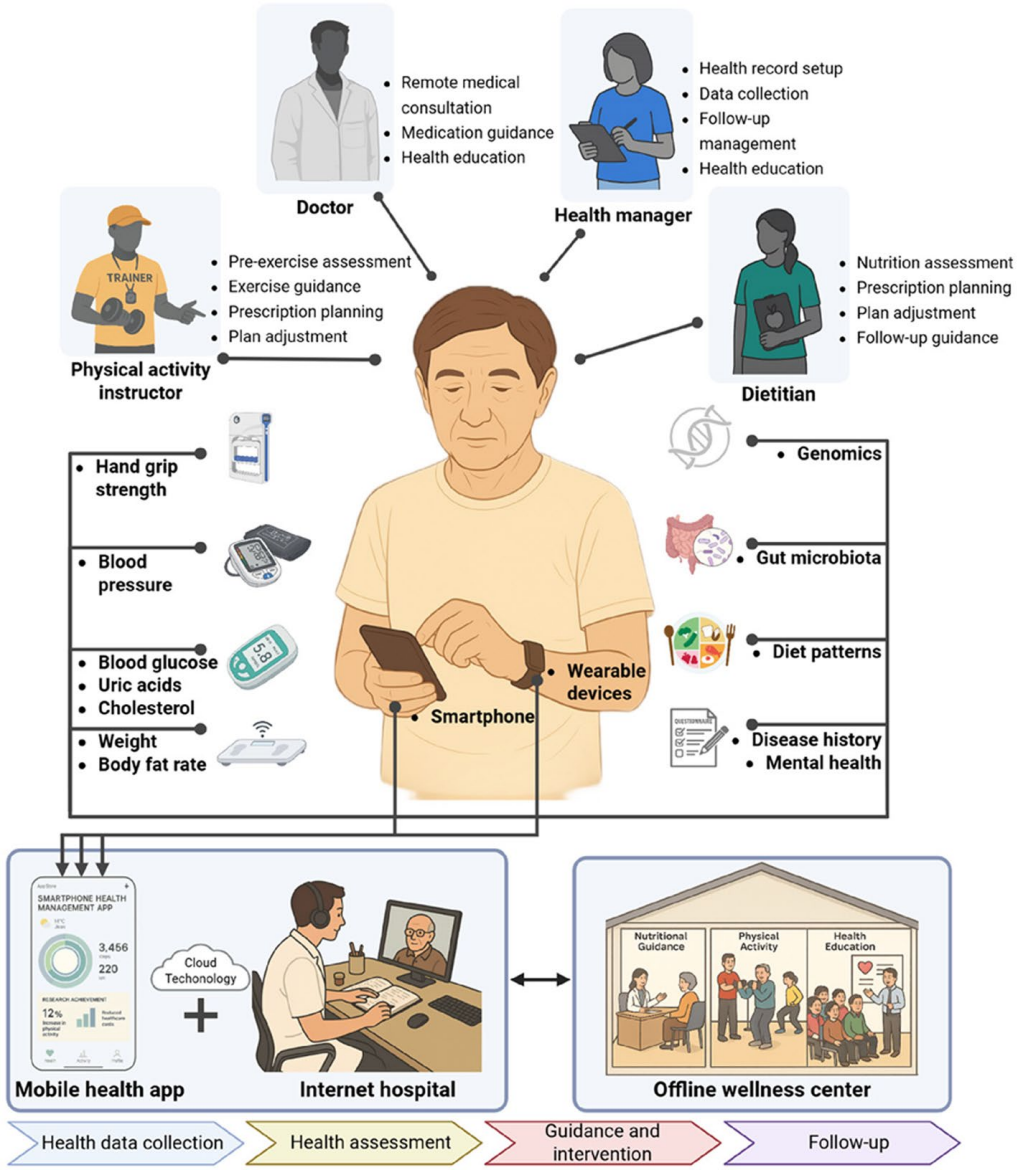
The multidomain lifestyle intervention framework. For each participant, the management group included at least four health managers, one dietitian, one physical activity instructor, and one doctor. During the daily health management, health data from wearable devices, questionnaires, and smart sensors have been collected and uploaded to the cloud-based platform. The specimen collection for gut microbiota and genomics is ongoing. The online digital platform is integrated with offline wellness centers to provide four steps of health management: health data collection, health risk assessment, personalized guidance and intervention, and long-term daily follow-up

The digital health platform connected participants with health managers, physical instructors, dietitians, and doctors to realize comprehensive health data collection, health risk assessment, and personalized guidance (Fig. 2). This platform included the mobile health (mHealth) app, risk management system, cloud computing platform, smart wearable devices, and other smart sensors. The majority of health management is administered by the digital health platform: (1) Health data collection. Wearable devices are recommended to be worn as long as possible to track daily physical activity, sleep characteristics, and heart rate. Home-use medical devices are used to track participants’ daily health status, including body composition (body mass index (BMI) and body fat percentage), BP, BG, blood lipids, and grip strength. Users could also upload their daily food logs and pictures of standardized meals through the app; (2) Health risk assessments. At baseline, participants completed a set of self-reported questionnaires within the mHealth app, covering demographics, medical and family history, and multidimensional well-being domains (Additional file 1). Based on this information, health managers provided an initial assessment of participants’ health risks. During follow-up, the app continuously monitored uploaded physiological measurements and triggered reminders when values fell outside normal ranges, serving as an additional real-time risk-monitoring mechanism. (3) Remote interventions. The digital health platform grouped and managed participants. Medication adherence reminders and clinically based digital coaching were incorporated to drive personalized dietary and lifestyle changes (detailed below). The dietitians or physical activity instructors regularly followed up with participants through the platform, providing health education and prompt feedback on the participants’ condition.

#### Internet hospital

Internet hospital services are also delivered through this program via the online platform. Before consultation, users needed to upload at least one official medical diagnosis from an offline institution. Afterwards, physicians conducted 20–30-min video consultations on the digital platform. They provide chronic disease counselling for the patient online based on uploaded electronic medical records (EMR), physical examination reports, and long-term health monitoring data. As completion of the consultation, physicians issue a visit summary and complete an EMR for each user.

#### Offline wellness center

This program has a network of over 200 offline wellness centers to support the adherence and continuity of health management. Each offline wellness center is staffed by at least one dietitian, one physical activity instructor, and four health managers. Dietitians are responsible for individualized nutritional assessment and guidance, and providing ongoing adjustments of dietary plans based on users’ changing health conditions. Physical activity (PA) instructors deliver on-site personalized exercise guidance and coaching, including both aerobics and resistance exercises. Health managers play a central coordinating role by establishing personal health records, collecting and managing users’ physiological and behavioral data, conducting regular follow-up visits, and delivering targeted health education. Though the digital health platform plays a central role in this framework, the integration of offline services complements the digital platform, enabling a continuum of multidomain care.

### Intervention

#### Physical activity intervention

The program implemented personalized and progressive interventions to achieve sustainable lifestyle modifications over short-term behavioral shifts and enhance physical capabilities (Additional file 1). Specifically, participants are recommended to complete at least 30 min of moderate-intensity aerobic training (e.g., brisk walking, cycling, stair climbing, dancing, or light running) or walk 8000 steps every day. Beginners are encouraged to start at lower intensities and gradually increase their goals. A hybrid intervention combining online and offline components was provided, in which the online component included (1) a wearable device for real-time activity and heart rate tracking and (2) a mobile health app for data aggregation and feedback. Related photos and other information (time, type, and duration) on daily physical activity are also uploaded to the mHealth app by participants. The offline component at wellness centers included (1) comprehensive physical fitness assessments conducted at baseline and quarterly to evaluate cardiorespiratory endurance, muscular strength, flexibility, and balance, (2) structured training plans developed and optimized by PA instructors, (3) 30-min group exercise sessions monthly with certified coaches maintaining participant engagement, ensuring proper technique, and providing support.

#### Dietary Intervention

Structured dietary intervention is implemented through a combination of an online platform and offline wellness centers. Each plan gives clear instructions on food selection (e.g., whole grains, low-sugar fruits, and lean proteins), portion sizes (e.g., ≤ 300 g grains and ≥ 200 g vegetables per meal, salt intake < 5 g/day), and meal sequencing (e.g., consumption of protein and/or fat before carbohydrate [17]). Based on the online digital platform, (1) daily dietary patterns are monitored by recording food logs and uploading standardized dietary plate photos through the mHealth app, (2) dietitians regularly review users’ dietary logs to develop and adjust personalized diet plans and targets, and (3) online guidance is also delivered to remind participants to follow a healthy diet. In offline wellness centers, dietitians provided quarterly workshops to introduce the principles of a healthy diet, such as balanced macronutrient distribution and hidden-sodium identification, thereby ensuring both dietary adherence and long-term behavior change.

### Measurements

#### Demographic data

Demographic data were collected when participants initially downloaded the app and included age, gender, smoking status, education, and geographic location***.*** Participants self-reported morbidities, including diabetes (Type 1 and Type 2 diabetes), hypertension, hyperlipidemia, coronary heart disease, fatty liver, stroke, myocardial infarction, chronic kidney disease, chronic obstructive pulmonary disease, asthma, arthritis, sleep disorders, and cancer.

#### Physiological metrics

Body composition (weight, BMI, and body fat percentage) was measured by a smart weight scale (CS20W), blood pressure was measured by BP monitors (C02L, Heal Force), blood glucose (daily glucose levels) and lipid profiles (total cholesterol) were measured by the GUC-1ble Glucose Meter. The monthly average value of body composition, BP, and BG was used as the primary outcome measure. The mean BG during the first month at baseline classified participants as: (1) Elevated blood glucose, BG of 10 mmol/L or greater [18]; (2) normal blood glucose, BG of 10 mmol/L or lower [18]. Similarly, elevated blood pressure was defined as either systolic BP ≥ 140 mmHg or diastolic BP ≥ 90 mmHg [19]. Obesity was defined as a body fat percentage of 30% or greater for males and of 42% or greater for females [20].

#### Behavioral and lifestyle data

Sleep characteristics (sleep duration, sleep timing, and deep sleep duration), physical activity (step counts and walking distance), and heart rate indices were tracked by Lexin Mio smart bracelet (Mambo, Lexin). The Mio smart bracelet is a wristband wearable device that utilizes photoplethysmography (PPG) and a three-axis acceleration sensor. Its validity has been reported previously [21]. Wearable devices underwent standard quality-control checks before assignment. This included device pairing, system time synchronization, and functional testing performed by trained staff. All participants were given specific instructions on its proper use and to wear it as long as possible. Device-generated data were automatically synced to the mHealth app.

#### Psychosocial assessments

A validated in-app questionnaire quantified emotional well-being (positive/negative affect scores) at baseline and quarterly intervals (Additional file 1).

#### Program engagement metrics

We characterized participant engagement using three measures. (1) Mobile health app usage was quantified as the weekly recording frequency across six main domains (blood pressure, blood glucose, body composition, physical activity, sleep, and heart rate). For each person-week, we counted the number of days in which ≥ 1 record was uploaded and calculated the average weekly usage frequency. This measure was categorized into tertiles (low, medium, and high engagement) and served as the primary proxy for engagement. We also clustered the weekly usage frequency across the follow-up duration into four classes using the latent class mixed models: low-stable, decreasing, increasing, and high-stable (Additional file 1). (2) Wearable device use was assessed as daily wear hours, which accounted only for the minutes reporting a valid heart rate measurement. As previously reported, a valid day was defined as a wear time of at least 5 h [22]. This helps minimize the bias caused by no or low smartwatch wear time [23]. (3) Binary indicator of offline participation, reflecting whether a participant attended any wellness-center consultation or coaching session during the follow-up period. This indicator was included in sensitivity analyses to capture an additional mode of program engagement.

### Statistical analysis

Descriptive statistics summarize demographics, clinical profiles, and physiological metrics at baseline stratified by age and gender. To meet basic completion, participants had to have ≥ 2 measurements in the first month. Continuous variables were reported as mean ± SD, and categorical variables were reported as frequencies (%).

We investigated the distribution of wearable device use (total time-worn, number of days per week worn, daily wear time) and health data uploads (total recording days, recording days per month, and max continuous days) stratified by different age and sex groups based on empirical values. Thus, age was categorized into four different age groups (age < 40, 45 ≤ age < 65, 65 ≤ age < 75, and age ≥ 75 years) after careful inspection of the distribution of age. To identify predictors of program engagement, multivariable linear regression models analyzed associations between daily wear time and covariates (age, gender, BMI, marital status, education status, region, season, presence of self-reported diabetes and hypertension).

We then developed a series of linear mixed-effects models to fit the trajectories of change in main outcomes (BP, BG, and body fat percentage). Given that participants enrolled in the program at different times, the duration of follow-up was not the same for all participants, we analyzed data from the baseline (1st month) to one year (the 12th month). The fixed effects included natural spline terms to capture nonlinear time trends and interaction terms between time and participants’ status at baseline (e.g., elevated BG vs. normal BG). A random intercept was included for each user to account for within-subject correlation. Model selection was guided by the Bayesian Information Criterion (BIC). Population-level systolic BP, BG, and body fat percentage trajectories were predicted across the first 12 months after recruitment. For the sensitivity analysis, we included recording months with at least four days of measurements.

We further assessed the relationships between engagement and changes in cardiometabolic health indicators (BP, BG, BMI, and body fat percentage) across the study period. Changes in these indicators were calculated relative to each participant’s median measurement value during the first month, which served as the baseline reference. Program engagement was calculated as the weekly frequency of mobile health usage and was categorized into tertiles (low/medium/high). Enrollment time-by-engagement interactions were incorporated to quantify dose–response relationships. Specifically, enrollment time is defined as the number of recording weeks from the start, and was transformed with a negative exponent to account for the non-linear time effect. A series of sensitivity analyses were performed to check the robustness of primary analyses: first, we used the trajectories of weekly app usage and offline participation (yes/no) as proxies of program engagement. Second, we restricted the analysis sample to participants with at least 12 months of follow-up to evaluate whether associations persisted beyond one year. Baseline was reset to the 12-month visit and engagement and physiological change values were computed from one year onward. Finally, we applied inverse probability of censoring weighting (IPCW) to the primary analysis to account for informative dropout during the follow-up (Additional file 1) [24].

To explore whether changes in body fat percentage could partially account for the associations observed between engagement and cardiometabolic outcomes, we conducted exploratory mediation analyses (Additional file 1). Age, gender, BMI, marital status, education, region, season, presence of diabetes, and hypertension were adjusted as covariates in all analyses. Missing outcome values were regarded as missing values in the mixed-effect model and were addressed using multiple imputations for variables with < 20% missingness.

Analyses were performed in R 4.1.0, with significance at *p* < 0.05 (two-tailed).

## Results

### Baseline characteristics

A total of 40,216 participants were enrolled between October 1, 2018, and Sep 9, 2024 across China (Fig. 1). 26,582 of the 40,216 participants were women (66.1%), and the median (IQR) age was 71 (64–75), with the majority (91.1%) over 40 years old (Table 1). Most participants were college or above, never smoked, married, and resided in eastern China. Among participants with clinical diagnosis information, 81.8% of participants have at least one disease. A wide range of diseases were reported, including hypertension (7273 [50%]), diabetes (4785 [32%]), and hyperlipidemia (3789 [26%]).

**Table 1:** Demographic characteristics and select baseline clinical diagnoses by age group

Variables	18–40 years	40–65 years	65–75 years	≥ 75 years
**Demographic (N = 40,216)**				
Sex				
Male	1520 (42%)	2272 (27%)	5696 (32%)	4146 (41%)
Female	2069 (58%)	6282 (73%)	12,327 (68%)	5904 (59%)
Education				
Junior high school or below	50 (6%)^a^	926 (31%)	2860 (38%)	1479 (32%)
High school	74 (8%)	953 (32%)	2147 (28%)	1261 (27%)
College or above	768 (86%)	1139 (37%)	2637 (34%)	1881 (41%)
No response	2697	5536	10,479	5429
Smoking status				
Never smoker	691 (84%)	2572 (89%)	5786 (88%)	3162 (92%)
Former smoker	12 (1%)	111 (4%)	350 (5%)	110 (3%)
Current smoker	116 (14%)	201 (7%)	428 (7%)	160 (5%)
No response	2770	5670	11,459	6618
Marital status				
Married	451 (46%)	2990 (89%)	6809 (84%)	3795 (80%)
Divorced, separated, or widowed	15 (1%)	321 (10%)	1197 (15%)	912 (19%)
Never married	521 (53%)	59 (1%)	59 (0.7%)	22 (0.4%)
No response	2602	5209	9958	5321
Area				
Eastern China	1957 (54%)	4483 (52%)	8736 (49%)	4226 (44%)
Midern China	719 (22%)	2470 (31%)	4904 (30%)	1998 (22%)
Western China	688 (24%)	1246 (17%)	3626 (21%)	3420 (34%)
No response	123	208	325	188
**Self-reported morbidities (N = 14,850)**				
Diabetes	49 (12%)	813 (30%)	2425 (35%)	1498 (36%)
Hypertension	58 (14%)	1026 (37%)	3568 (50%)	2621 (61%)
Hyperlipidemia	30 (8%)	619 (23%)	2015 (30%)	1125 (29%)
Coronary heart disease	17 (4%)	241 (9%)	1051 (16%)	754 (19%)
Fatty liver	4 (5%)	202 (9%)	632 (11%)	319 (9%)
Stroke	9 (2%)	94 (4%)	428 (6%)	308 (8%)
Myocardial infarction	8 (4%)	70 (3%)	245 (4%)	185 (5%)
Chronic kidney disease	7 (2%)	78 (3%)	307 (5%)	260 (7%)
Chronic obstructive pulmonary disease	0 (0%)	10 (0.3%)	39 (0.6%)	41 (1%)
Asthma	14 (4%)	34 (1%)	93 (2%)	42 (1%)
Arthritis	12 (3%)	316 (12%)	1161 (17%)	682 (17%)
Sleep disorder	1 (1%)	124 (6%)	503 (9%)	317 (9%)
Cancer	8 (2%)	69 (3%)	207 (3%)	127 (3%)
**Number of Multimorbidity (N = 10,203)**				
0	32 (52%)	479 (26%)	884 (17%)	465 (15%)
1	17 (27%)	557 (30%)	1416 (28%)	864 (27%)
2	5 (8%)	401 (22%)	1172 (23%)	729 (23%)
≥ 3	8 (13%)	393 (21%)	1635 (32%)	1146 (36%)

Continuous variables are presented as mean (SD) and categorical variables are shown as number (percentage)

Among 15,548 participants with valid heart rate data (Additional file 2: Fig. S1), they wore their watches on a median of 463 days (IQR 55–714) (Additional file 2: Table S2), and the median daily watch wear time was 17.7 h (IQR 11.6–20.2). 14,805 (95.5%) participants wore their watches for 8 h or longer, and 11,395 (73.5%) for 12 h or longer a day. Over the management period, 29,030 participants generated 9,749,898 physical activity measurements with 335.9 (SD: 458.4) per participant. Completed information about the data uploads in each module is listed in Additional file 2: Table S1.

Physiological health metrics varied by age and gender (Additional file 2: Table S3). Males exhibited higher body mass index (BMI: 24.1–25.1 kg/m^2^ vs. 23.7–24.8 kg/m^2^) and lower body fat percentage compared to females (30.3–35.2% vs. 24.5–25.6%). BP indices showed age-related increases, with systolic BP rising from 112.5–123.6 mm Hg (18–40 years) to 130.5–131.2 mm Hg (≥ 75 years). Heart rate declined with age, with daily averages decreasing from 78.7–79 beats/min (18–40 years) to 72.7–73.4 beats/min (≥ 75 years). Glycemic control indices revealed higher BG levels in males (7–7.9 mmol/L vs. 6.6–7.7 mmol/L in females). Muscle strength, measured by hand grip strength, declined with age and was consistently higher in males (36.1–42.9 kg vs. 28.4–36.8 kg in females). Sleep duration increased with age, with total sleep time rising from 402–411.4 min (18–40 years) to 421.5–428.5 min (≥ 75 years). Females exhibited longer deep sleep duration (108.4–125.3 min vs. 90.6–108.7 min in males). Physical activity peaked in the 65–75 age group (7,347.3–7,469.3 daily steps), declining thereafter. Interestingly, positive emotion scores increased marginally with age (17.5–19.3 in males; 18.1–19.0 in females), while negative emotion scores decreased (12.1–9.2 in males; 11.2–9.4 in females).

### Wearable device usage and health data recordings across age groups and gender

Figure 2 shows that wearable device usage varies by age and gender (Additional file 2: Table S4). Older participants exhibited longer total wear time, while women generally wore the devices longer than men across all age groups. The average number of valid wearing days (> 5 h) per week ranges from approximately 4 to 5 days across age groups. Daily wear time increased with age, with older participants showing higher adherence. For those above 40, they wore the watch for over 15 h per day.

**Fig. 3:**
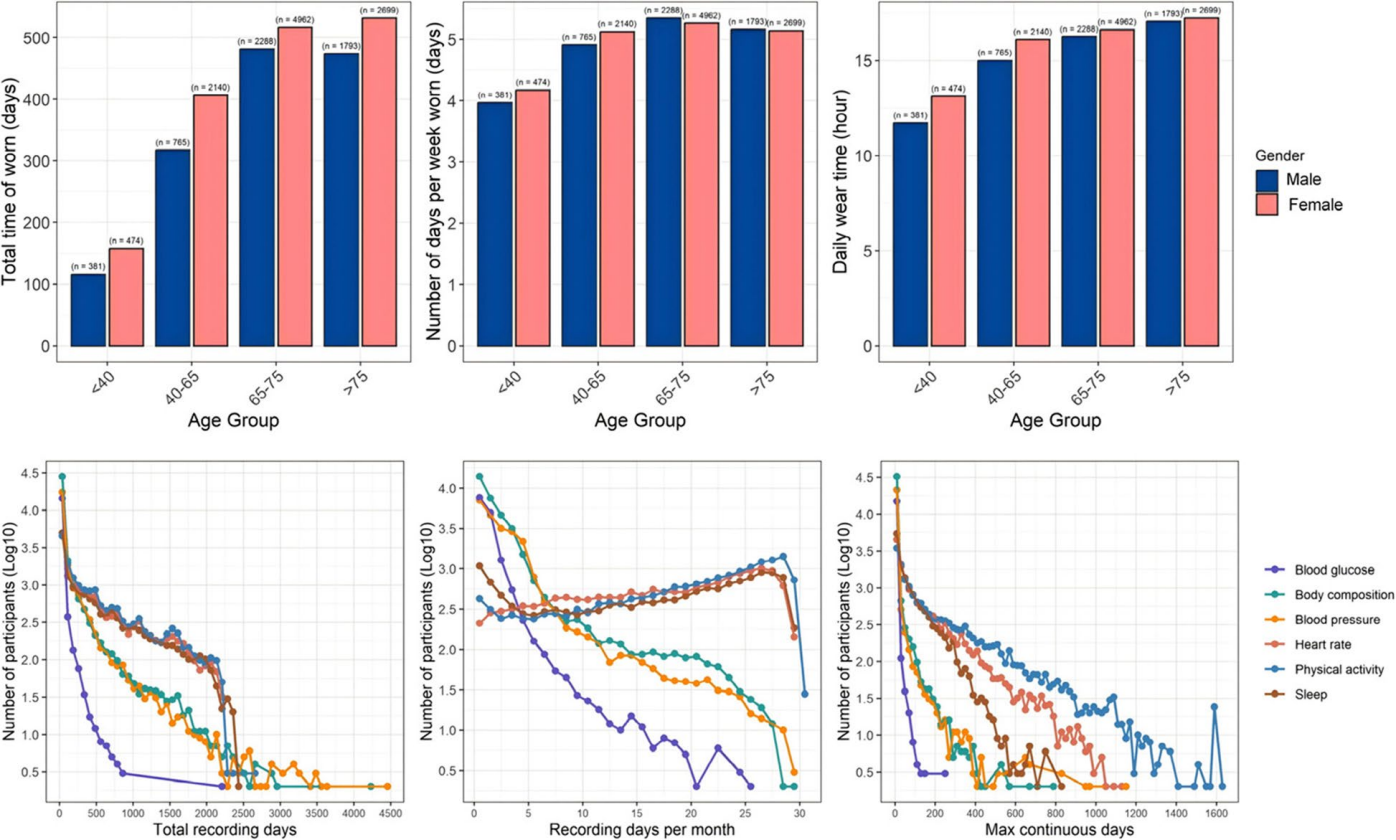
Characteristics of wearable device use and health data uploads. **A** Wearable device usage varies by age and gender. Wearable device usage was characterized by total time worn, number of days per week worn, and daily wear time; **B** Distribution of health data recordings in six main datasets in the program. The data recording characteristics of each dataset per participant were classified as total recording days, recording days per month, and maximum continuous recording days

The distribution of total recording days, monthly recording days, and maximum continuous recording days from other health databases is also depicted in Fig. 3. The overall distribution followed a logarithmic trend, except for metrics (physical activity, heart rate, and sleep) measured by wearable devices, which exhibited the highest long-term engagement. Among different physiological parameters, physical activity, heart rate, and BP had the most sustained long-term data collection, with some participants recording data for over 1,500 consecutive days.

### Relationships between sociodemographic characteristics and engagement

Multivariable linear regression was used to model the relationship between sociodemographic factors and engagement, as measured by weekly frequency of mobile health app usage and daily device wear time (hours). Several predictors were identified as significantly associated with engagement (Table 2). Age (*β* = 0.05, 95% CI: 0.05 to 0.05; *β* = 0.01, 95% CI: 0.01 to 0.01) was associated with both longer device wear time and more weekly usage. In comparison, gender, BMI categories, and geographical regions showed inconsistent associations. Female gender (*β* = 0.31, 95% CI: 0.15 to 0.47) was associated with longer daily wear time but showed no association with weekly usage of the mobile health app. Compared to participants with a normal BMI, a higher BMI was associated with longer wear time but lower weekly health recording uploads. Geographical disparities persisted; compared to participants living in western China, those living in mid- and eastern China demonstrated shorter wear time but a higher frequency of app usage. Notably, disease status, such as diabetes and hypertension, didn’t demonstrate significant associations with wearable use, but hypertension was associated with more mobile health app usage.

**Table 2:** Multivariable analysis of engagement and sociodemographic characteristics

Predictors	Differential weekly app usage (95% CI), day^a^	*P* value	Differential daily wear time (95% CI), h	*P* value
Age	0.01 (0.01 to 0.01)	< 0.001	0.05 (0.05 to 0.05)	< 0.001
Gender				
Male	1 [Reference]	1 [Reference]	1 [Reference]	1 [Reference]
Female	0.06 (−0.02 to 0.14)	0.109	0.31 (0.15 to 0.47)	< 0.001
BMI categories				
Underweight	−0.05 (−0.09 to −0.01)	0.015	0.87 (0.81 to 0.93)	< 0.001
Normal	1 [Reference]	1 [Reference]	1 [Reference]	1 [Reference]
Overweight	−0.09 (−0.11 to −0.07)	< 0.001	0.04 (0.02 to 0.06)	< 0.001
Obese	−0.21 (−0.25 to −0.17	< 0.001	0.18 (0.12 to 0.24)	< 0.001
Marital status				
Married	1 [Reference]	1 [Reference]	1 [Reference]	1 [Reference]
Never married	−1.17 (−1.46 to −0.88)	< 0.001	−1.82 (−2.53 to −1.11)	< 0.001
Divorced, separated, widowed	0.28 (0.18 to 0.38)	< 0.001	0.03 (−0.17 to 0.23)	0.760
Education level				
Junior high school or lower	1 [Reference]	1 [Reference]	1 [Reference]	1 [Reference]
High school	0.34 (0.26 to 0.42)	< 0.001	0.13 (−0.07 to 0.33)	0.179
College or above	0.49 (0.41 to 0.57)	< 0.001	0.07 (−0.11 to 0.25)	0.429
Geographical region				
West	1 [Reference]	1 [Reference]	1 [Reference]	1 [Reference]
Mid	1.52 (1.42 to 1.62)	< 0.001	−0.74 (−0.96 to −0.52)	< 0.001
East	1.42 (1.32 to 1.52)	< 0.001	−0.9 (−1.12 to −0.68)	< 0.001
Season				
Spring	1 [Reference]	1 [Reference]	1 [Reference]	1 [Reference]
Summer	−0.08 (−0.08 to −0.08)	< 0.001	−0.18 (−0.2 to −0.16)	< 0.001
Autumn	−0.08 (−0.08 to −0.08))	< 0.001	−0.2 (−0.22 to −0.18)	< 0.001
Winter	−0.3 (−0.3 to −0.3)	< 0.001	−0.1 (−0.12 to −0.08)	< 0.001
Diabetes	−0.06 (−0.14 to 0.02)	0.118	0.12 (−0.04 to 0.28)	0.139
Hypertension	0.11 (0.03 to 0.19)	0.001	0 (−0.16 to 0.16)	0.999

^a^The frequency of mobile health app usage was calculated as days of health data recordings across six datasets (BP, BG, heart rate, body composition, physical activity, and sleep characteristics) per week

### BP, BG, and body fat percentage change over time

**Fig. 4:**
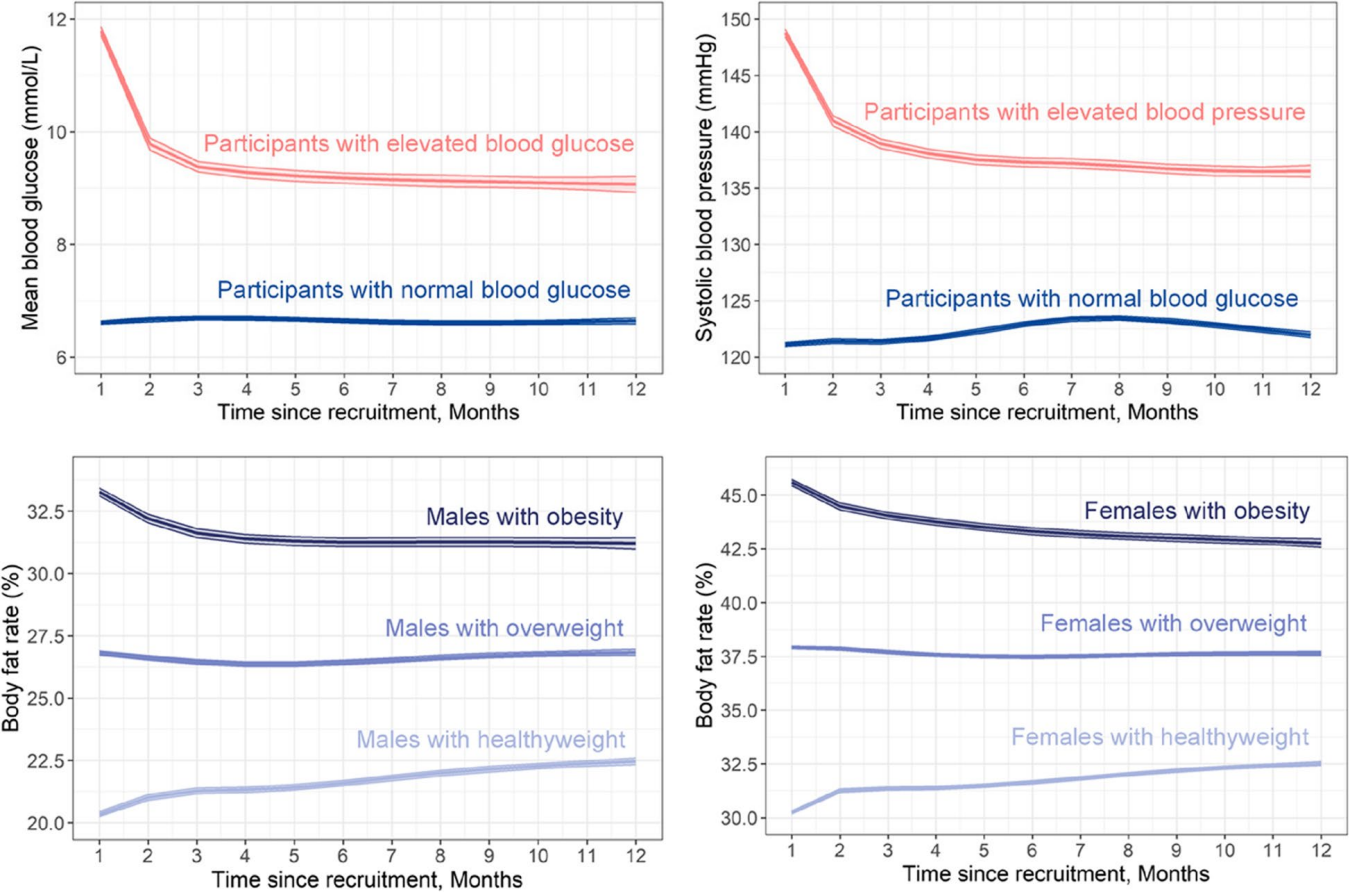
BG, BP, and body fat percentage change over a 12-month follow-up. BG, blood glucose; BP, blood pressure. Elevated BG was defined as a BG of 10 mmol/L or higher; Elevated BP was defined as either systolic BP ≥ 140 mmHg or diastolic BP ≥ 90 mmHg; Obesity was defined as body fat percentage ≥ 30% for males and ≥ 42% for females; Overweight was defined as body fat percentage ≥ 25% for males and ≥ 36% for females

Figure 4 presents population-level BP, BG, and body fat percentage change over a 12-month follow-up. Linear mixed effects model was applied to fit the trajectory stratified by baseline health conditions (elevated blood glucose, elevated blood pressure, obesity). Participants with elevated BG levels at baseline exhibited a sharp decline in mean BG from around 12 mmol/L to under 10 mmol/L within the first two months, followed by a gradual stabilization over time. In contrast, normoglycemic participants maintained relatively stable glucose levels (~ 6.5–7.0 mmol/L) throughout the study. Similarly, among participants who started with elevated BP and obesity, systolic BP (from ~ 150 mmHg to ~ 138 mmHg) and body fat percentage (males: ~ 34% to ~ 32%; females: ~ 46% to ~ 44%) demonstrated a rapid reduction within the initial months. Individuals with overweight exhibited a relatively stable body fat percentage, whereas healthy-weight individuals experienced a slight increase in body fat percentage over the study period. Additional file 2: Table S5 details the proportions of improvement among participants with elevated BG, BP, and body fat percentage at baseline. For example, among participants who started with elevated BG, 77%, 80%, 83%, and 85% of participants improved their BG control after 2, 4, 6, and 12 months. A sensitivity analysis including at least 4 measurements for each month revealed a similar pattern (Additional file 2: Fig. S2).

### Associations between BP, BG, body composition, and program engagement

Greater engagement with the health management program was associated with lower SBP (Table 3). For example, among participants with elevated BP, high- and medium-engagement were associated with significant decreases in systolic BP over time (medium-engagement: −1.98 mm Hg; 95% CI, −3.37 to −0.59 mm Hg; high-engagement: −3.85 mm Hg; 95% CI, −5.2 to −2.49 mm Hg). Effect modification by baseline BP status was observed (Additional file 2: Table S6). Higher engagement levels among non-hypertensive participants were slightly associated with a decrease in SBP over 12 months (−0.76 mmHg, 95% CI: −1.41 to −0.11). A declining time trend with follow-up weeks for SBP and DBP was also observed in participants with elevated BP.

**Table 3:** Adjusted Associations Between Blood Pressure, Blood Glucose, Body Composition, Engagement, and Time Since Enrollment

Health indicators	Time change^e^	Medium (vs. Low) engagement	High (vs. Low) engagement	Medium engagement × Time change	High engagement × Time change
**Change in Systolic Blood Pressure**^**a**^					
Participants with elevated blood pressure	**9.75 (8.46, 11.04)**^**f**^	**−1.82 (−3.17, −0.46)**	**−3.65 (−4.97, −2.33)**	**2.39 (0.98, 3.81)**	**6.68 (5.34, 8.02)**
Participants with normal blood pressure	**−0.86 (−1.52, −0.21)**	−0.11 (−0.77, 0.54)	**−0.68 (−1.32, −0.04)**	0.46 (−0.25, 1.16)	**1.5 (0.82, 2.17)**
**Change in Diastolic Blood Pressure**					
Participants with elevated blood pressure	**4.39 (3.59, 5.18)**	−0.39 (−1.24, 0.47)	−0.73 (−1.56, 0.11)	0.68 (−0.19, 1.55)	**1.74 (0.92, 2.57)**
Participants with normal blood pressure	−0.04 (−0.48, 0.4)	−0.04 (−0.49, 0.4)	−0.2 (−0.63, 0.24)	0.18 (−0.3, 0.65)	0.39 (−0.06, 0.84)
**Change in Blood Glucose**^**b**^					
Participants with elevated blood glucose	**2 (1.11, 2.89)**	−0.12 (−0.99, 0.75)	**−1.21 (−2.07, −0.35)**	0.42 (−0.5, 1.33)	**1.52 (0.62, 2.42)**
Participants with normal blood glucose	−0.06 (−0.26, 0.15)	0 (−0.19, 0.2)	−0.01 (−0.2, 0.18)	0.14 (−0.07, 0.35)	**0.26 (0.05, 0.46)**
**Change in Body fat percentage**^**c**^					
Participants with obesity	**1.07 (0.74, 1.39)**	−0.32 (−0.69, 0.04)	**−1.25 (−1.61, −0.89)**	**0.42 (0.05, 0.8)**	**1.84 (1.48, 2.2)**
Participants with overweight	**0.26 (0.13, 0.39)**	0 (−0.14, 0.13)	**−0.54 (−0.67, −0.41)**	−0.04 (−0.2, 0.12)	0.75 (0.6, 0.9)
Participants with healthy weight	**−0.87 (−1.03, −0.7)**	**0.95 (0.73, 1.16)**	**1.75 (1.54, 1.95)**	**−1.02 (−1.21, −0.82)**	**−1.73 (−1.91, −1.55)**
**Change in BMI**^**d**^					
Participants with obesity	**0.8 (0.55, 1.04)**	**−0.27 (−0.55, 0)**	**−0.63 (−0.89, −0.36)**	**0.54 (0.26, 0.81)**	**1.11 (0.84, 1.37)**
Participants with overweight	**0.34 (0.27, 0.41)**	**−0.14 (−0.22, −0.07)**	**−0.49 (−0.56, −0.42)**	**0.21 (0.13, 0.29)**	**0.76 (0.69, 0.84)**
Participants with normal weight	−0.03 (−0.08, 0.03)	**0.23 (0.16, 0.3)**	**0.53 (0.46, 0.6)**	**−0.16 (−0.22, −0.09)**	**−0.3 (−0.36, −0.24)**
Participants with underweight	**−0.46 (−0.71, −0.2)**	**0.69 (0.37, 1.01)**	**0.8 (0.48, 1.13)**	**−0.71 (−1, −0.41)**	**−1.15 (−1.43, −0.86)**

^a^ Elevated BP was defined as either systolic BP ≥ 140 mmHg or diastolic BP ≥ 90 mmHg; ^b^ Elevated BG was defined as a BG of 10 mmol/L or higher; ^c^ Obesity was defined as body fat percentage ≥ 30% for males and ≥ 42% for females; Overweight was defined as body fat percentage ≥ 25% for males and ≥ 36% for females. ^d^Obesity was defined as BMI ≥ 30 kg/m^2^; Overweight was defined as 30 kg/m^2^ ≥ BMI > 25 kg/m^2^; Normal weight was defined as 25 kg/m^2^ ≥ BMI > 18.5 kg/m^2^.^e^Time was transformed with a negative exponent to account for the non-linear time effect, calculated as exp(-time since start in weeks). A positive coefficient represents a decreasing trend for the change in cardiometabolic metrics. ^f^parenthetical values represent 95% confidence intervals

Participants with elevated BG exhibited a significant decrease in BG levels over time (Table 3). Among them, high engagement was associated with a greater reduction (−1.2 mmol/L, 95% CI: −2.07 to −0.35). High engagement also showed a significant interaction with follow-up weeks, indicating that participants with high engagement experienced a steeper decline in blood glucose over the follow-up period. Participants with obesity showed a similar decreasing association pattern in body fat percentage over time, as high engagement (−1.25%, 95% CI: −1.61 to −0.89) was linked to a greater reduction in body fat among obese individuals, whereas overweight and healthy-weight individuals exhibited smaller changes. Sensitivity analyses based on engagement trajectory classes (Additional file 2: Table S7) and offline participation (Additional file 2: Table S8) demonstrated attenuated but consistent associations. Given the heterogeneity in adherence and informative dropout during the follow-up, we also performed IPCW-weighted associations (Additional file 2: Table S9) and analyses among participants with over one year of follow-up (Additional file 2: Table S10). These sensitivity analyses showed estimates similar to the primary results.

We investigated whether the associations of engagement with BP and BG might be mediated by changes in participant BMI or body fat percentage. Exploratory mediation analysis showed that body fat percentage mediated the association between engagement and reduction in BP and BG (Additional file 2: Table S11). For example, we found that in overweight participants, high engagement has both direct and indirect effects on reduction in BG (indirect: −0.006 mmol/L, 95% CI: −0.01 to −0.004 mmol/L; direct: −0.124 mmol/L, 95% CI: −0.204 to −0.046 mmol/L) and SBP (indirect: −0.015 mmol/L, 95% CI: −0.019 to −0.011 mmol/L; direct: −0.115 mmol/L, 95% CI: −0.173 to −0.055 mmol/L).

## Discussion

China is taking steps to build up institutional and community care infrastructure as a complement to traditional family care, where digital health plays a central role in this transition [3]. Though digital medicine technology has been widely applied to encourage the self-management of older adults’ health [25], real-world evidence has been scarce to help the transition from research into public health practice. Most previous digital health research often uses only 3 to 7 daily measures during a snapshot period [26–29]. Short monitoring periods are prone to an observer effect and may not accurately reflect true short- and long-term activity behavior [30], which calls for long-period real-world assessments. Nevertheless, observational studies with longitudinal long-term tracking often focus on a specific and narrow set of health assessments, like menstrual cycle [31], sleep characteristics [32], or solely on BP [23, 33]. In comparison, real-world digital health interventions typically measure multidimensional information, including clinical, behavioral, and physiological data, but these intervention programs were only applicable to a small number of participants [9, 10, 34, 35]. In 2022, Golbus et al. started a framework that collected dense digital data integrated with other measures of health (e.g., electronic health records (EHRs)) to generate a longitudinal repository for a diverse patient population, but the associations between engagement and health outcomes within this framework have not yet been investigated [36]. Therefore, to the best of our knowledge, this is the first study reporting a real-world implementation of a health management program for cardiometabolic health that has achieved substantial population coverage and maintained high engagement throughout the study period.

Long-term adherence and engagement are well-known challenges in digital health studies [37]. For example, a recent Framingham Heart Study remote-monitoring report documented one-year retention of 44% for smartwatch use and 21% for blood-pressure monitoring[23]. Similarly, in the Adolescent Brain Cognitive Development Study, app-based wearable engagement declined to approximately 75% within the first month [38]. In the MyHeart Counts study via a smartphone app, investigators reported that 47% of the participants completed just 2 consecutive days of fitness measurements in the first week, and the engagement declined afterwards [26]. In this context, the frequency of mobile health app use per week in the current study, as well as the retention rate, did not appear to be lower and may have been even more successful. This result could support the perspective that large-scale adoption of home health care requires a good mixture of baseline training, reminders, feedback, and participants’ flexibility [39]. We suggest that digital health-based studies should incorporate different sources of health data to facilitate clinical recommendations and public health interventions for a broader population.

Previous studies of mobile technology–facilitated self-management interventions have shown that it is useful to modulate healthy lifestyles (e.g., diet [40] and physical activity [41]) to improve cardiometabolic health (e.g., weight [42], BP [43], BG [44], and lipid profile [45]). For example, Gazit et al. showed that by employing digital health, participants with elevated BP could have at least a 7.2 mmHg reduction in systolic BP over 3 years [8]. Moreover, evidence showed that digital health platforms that focus on BP monitoring could lead to blood glucose reduction [9]. The present study’s findings are consistent with these studies. Notably, we demonstrated that among the participants with cardiometabolic risk at baseline, high engagement (vs. low engagement) was associated with considerable improvement in BP, BG, and body fat percentage (reductions in 3.9 mmHg, 1.2 mmol/L, and 1.2%). These results are noteworthy in the context of earlier evidence, which showed that improvements in cardiometabolic metrics, such as BP [46], BG [47, 48], and body fat percentage [49], are associated with lower risks of primary endpoints at the population level. For instance, reductions in SBP by 1 mm Hg were associated with a 4% decrease in the incidence of stroke and a 2% decrease in coronary heart disease events [46]. Given the BP reduction magnitudes observed in our study, these associations underscore the potential role of digital health in supporting community-based aging care programs. However, it should also be noted that our findings are based on individuals who engaged with a commercial digital-health program, and systematic differences in social and behavioral characteristics may exist between these users and the wider aging population. Therefore, the observed benefits of engagement and their generalizability should be interpreted with caution.

Our study has multiple strengths. One primary strength of our study is integrating digital health technologies and offline wellness centers. Based on this framework, a multidomain management procedure was provided, encompassing continuous health data monitoring, lifestyle interventions, internet hospital, and health education, which enhances the engagement and utility of this program. In comparison, conventional mHealth interventions often rely solely on mobile apps or single-component interventions, which may limit sample adherence and sustained engagement. For example, the MyHeart Counts smart app was utilized to monitor physical activity patterns in a 7-day study, but only 11.1% of participants completed consecutive 7 days of recording [26]. Additionally, interventions based solely on text messages showed mixed results, as they may fail to improve cardiovascular health [50]. As such, it has been demonstrated that providing personalized and multidimensional interventions could increase adherence and effects [12, 51–53]. One recent study showed that internet-delivered multidomain lifestyle intervention could result in significantly better cognition in older adults over 3 years [54]. The present study extends this structured research by incorporating offline wellness centers in real-world settings. Secondly, another key strength of our study is its geographically diverse sample, which enhances the generalizability of our findings. Our study includes over 40,000 participants across 30 provinces, covering northeastern, eastern, central, and western China. We demonstrated a higher proportion of participants resided in Eastern China, consistent with previous evidence, which showed that digital health use is associated with higher education level and household income [23, 55]. Nevertheless, the high engagement rates across nearly all provinces and even higher wearable use in Western China support the idea that digital health might reduce geographical inequalities by improving older adults’ access to healthcare services [3, 56]. Third, multiple cardiometabolic health metrics integrated with demographic and disease information were collected longitudinally. In comparison, most of the previous studies have deeply phenotyped participants over shorter periods or collected data for only one endpoint [57–59]. Fourth, the present real-world study is highly pragmatic, which supports the feasibility of digital health-based management among large-scale aging populations. Nevertheless, deeper investigations into the mechanisms underlying the observed associations with cardiometabolic health indicators are warranted.

Our study also has several important limitations. First, the observational nature of our data precludes a definitive causal interpretation of our findings. More rigorous study designs, such as randomized controlled trials (RCTs), will be needed in future research to further clarify these relationships. Second, the participants comprised only users who participated in the commercial health management program. Digital participation is unequally distributed among older adults, as those with disadvantaged backgrounds have more limited access to digital technologies [60]. Thus, selection bias may exist that limits the generalizability of our results to non-retained individuals. Third, we also cannot exclude the possibility of selection bias due to the loss of follow-up, since not all participants remained engaged in the program over time. Healthier adherers may have been more likely to continue in the program long term, which may introduce heterogeneity in outcomes. Fourth, though a series of covariates were adjusted, we cannot entirely rule out the possibility of unmeasured confounding factors. For example, our data does not allow for precise quantification of the individualized guidance provided by dietitians and fitness coaches, which might introduce unmeasured variability in the observed associations. Fifth, measurement errors may be present, given that individuals need to self-report their BG, BP, and body composition values, which might differ from clinic measures. Additionally, while PPG and accelerometer-based wearable devices enable large-scale, continuous monitoring in real-world settings, their measurements may contain greater noise and device-specific bias than validated research-grade sensors. Furthermore, despite multiple imputations, variations in follow-up durations and incomplete records may affect the robustness of longitudinal analyses.

## Conclusions

In this study, we introduce a large-scale digital health practice in China. Participants in this digital-health-based multi-domain lifestyle management achieved long-term control of cardiometabolic health. This real-world evidence suggests that digital health may be useful and scalable for daily cardiometabolic health monitoring and management.

## Supplementary Information

Additional file 1: Supplementary method of data collection procedure, contents of the intervention measures, health risk assessment scale, questionnaire for psychological assessment, and statistical analyses

Additional file 2: Table S1. The number of total health recordings and participants in each of the eight main datasets in the program; Table S2. Persistent use for the first year in the program; Table S3. Physiological, psychological, and lifestyle data by age and gender group at baseline; Table S4. Wearable use of participants across age and sex group; Table S5. Blood glucose, blood pressure, and body fat rate reduction over time by initial health status at baseline; Table S6. Associations Between Cardiometabolic Health and the Interactions of Baseline Status, Engagement, and Time Since Enrollment; Table S7. Adjusted Associations Between Blood Pressure, Blood Glucose, Body Composition, Engagement trajectories, and Time Since Enrollment; Table S8. Adjusted Associations Between Blood Pressure, Blood Glucose, Body Composition, Offline Participation, and Time Since Enrollment; Table S9. Adjusted Associations Between Blood Pressure, Blood Glucose, Body Composition, Engagement, and Time Since Enrollment, after inverse-probability-of-censoring weighted; Table S10. Adjusted Associations Between Blood Pressure, Blood Glucose, Body Composition, Engagement, and Time Since Enrollment, restricting to participants with over one year follow-up; Table S11. Mediating analysis of program engagement on BP, and BG through the change in body fat percentage; Figure S1. Flow chart of the study design; Figure S2. BG, BP, and body fat percentage change over 12-month follow-up with each month having at least four measurements

## Data Availability

The datasets used and/or analyzed during the current study are available from the corresponding author upon reasonable request.

## Abbreviations

IQR: Interquartile range
BP: Blood pressure
BG: Blood glucose
RWE: Real-world evidence
mHealth: Mobile health
BMI: Body mass index
EMR: Electronic medical records
PA: Physical activity
PPG: Photoplethysmography
IPCW: Inverse probability of censoring weighting
SD: Standard deviation
SBP: Systolic blood pressure
CI: Confidence interval
DBP: Diastolic blood pressure
EHRs: Electronic health records
RCTs: Randomized controlled trials
