# Supplementary material for: A digital-health multidomain lifestyle management framework and its associations with cardiometabolic health: a real-world observational study
Source: BMC Med. 2026 Mar 25;24:285. doi: 10.1186/s12916-026-04830-y (PMC13137710; doi:10.1186/s12916-026-04830-y)
Supplement: Supplementary file 1 — Additional file 1: Supplementary method of data collection procedure, contents of the intervention measures, health risk assessment scale, questionnaire for psychological assessment, and statistical analyses [file 12916_2026_4830_MOESM1_ESM.docx]

**Supplementary Method**

1. **Data collection procedure**

***Body composition***

Body composition was assessed using digital body composition scale. Participants were instructed to fast for at least 2 hours prior to measurement and to avoid intense physical activity, alcohol, and diuretics during this time. Female participants were advised to avoid testing during menstruation. Participants wore light clothing and removed metal accessories to minimize interference. After cleaning the skin-electrode contact points with electrolyte wipes, participants stood barefoot on the device, feet shoulder-width apart, and held the hand electrodes with arms naturally extended. After maintaining a stable posture for 5 minutes, the measurement was initiated. Individuals with implanted metal devices or severe edema were excluded from bioelectrical impedance analysis (BIA) testing.

***Blood Glucose and Blood lipids***

Capillary blood glucose was measured using Blood Glucose, Uric Acid, and Cholesterol Tester (GUC-1). Participants washed their hands with warm water and soap and disinfected the puncture site with alcohol, ensuring complete evaporation before sampling. Participants took their measurements at varying time in daily life: Fasting blood glucose was measured after 8–10 hours of fasting; postprandial blood glucose was measured 2 hours after the first bite of a meal. Blood samples were obtained from the lateral side of the fingertip (preferably the ring or middle finger), avoiding areas with wounds or swelling. The first drop of blood was wiped away, and the second drop was applied to the test strip. Readings were uploaded to the app, and all materials were disposed of according to medical waste protocols. Participants were advised to remain calm before testing, as emotional stress, recent exercise, and medications may influence glucose levels.

***Blood Pressure***

Blood pressure was measured using digital blood pressure cuff. Participants avoided caffeine, alcohol, smoking, and exercise for at least 30 minutes before the test and rested in a quiet environment for 5–10 minutes. Measurements were performed with the participant seated, feet flat on the floor, back supported, and the arm resting at heart level. The cuff was positioned 2–3 cm above the elbow crease, snug but not overly tight. Two to three readings were taken at 1–2 minute intervals, and the average value was recorded.

***Handgrip Strength***

Grip strength was measured using a digital handgrip dynamometer. Participants activated the device by shaking it, then stood upright with arms at their sides. They were instructed to grip the device maximally for at least 6 seconds. The measurement was completed when data were automatically uploaded to the system.

1. **Contents of the intervention measures**

| **Daily health tracking** | |  |
| --- | --- | --- |
| 1 | Health record establishment on the mHealth app and health information collection, including demographics, disease history, and family history | Health managers |
| 2 | Physical examination and health assessment at the baseline | Health managers |
| 3 | Wear a smart wearable device to monitor physical activity, sleep characteristics, and heart rate | Participants |
| 4 | Measure the blood pressure, blood cholesterol, and fingertip blood glucose every week and upload the data to the app | Participants |
| 5 | Record the body weight, body fat rate, bone density, and lean mass rate every day and upload the data to the app | Participants |
| 6 | Fill a self-designed 5-point mental health questionnaire and upload the data to the app | Participants |
| 7 | Collect medication information of participants with abnormal blood pressure or blood glucose and provide regular medication reminders | Health managers |
| **Remote dietary intervention** | |  |
| 1 | Provide one-on-one personalized dietary guidance to the participants via the mHealth , based on factors such as age, gender, weight, food intake, chronic disease status, and portion size (3–5 times a week) | Registered dietitians |
| 2 | Follow the personalized dietary principles and upload the food logs and photos of everyday dietary plates to the mHealth app | Participants |
| 3 | Provide monthly consultations to review users’ dietary logs, then develop and adjust personalized diet plans and targets | Registered dietitians |
| 4 | Provide web-based health education about the importance of weight management and benefits of reasonable dietary intake | Registered dietitians |
| 5 | Establish a WeChat management social group including participants to remind and urge them to upload and record daily dietary intake information | Health managers |
| **Remote physical activity intervention** | |  |
| 1 | Evaluate the physical-fitness of participants at baseline and every quarter | Physical activity instructors |
| 2 | Wear a wearable device connected to the mHealth app and upload daily walking step counts; other physical activities should be recorded on the web and uploaded to the app | Participants |
| 3 | Design personalized training programs and provide one-to-one guidance sessions | Physical activity instructors |
| 4 | Lead group exercise classes on-site monthly (30 minutes per session) and continuously monitor users’ attendance, performance, and feedback, adjusting exercise plans to optimize physical outcomes | Physical activity instructors |
| 5 | Provide web-based health education about the importance of weight management and the benefits of regular physical activity (resistance exercise and aerobic exercise) | Physical activity instructors |
| 6 | Establish a WeChat management social group to remind and urge participants to upload and record daily physical activity information | Health managers |

1. **Health status assessment**

Health risk in the app was evaluated using a multidimensional self-reported assessment covering five domains of general well-being rather than cardiometabolic risk specifically. The assessment incorporated validated items across:

1. General Health Perception (GHP): 3 items from the SF-36 assessing subjective overall health;
2. Physical Functioning (PF): 9 SF-36 items evaluating limitations in physical activity and daily living activities;
3. Psychological Well-Being (PWB): 8 SF-36 items capturing positive and negative affect;
4. Resilience (RES): 6 items from the Connor–Davidson Resilience Scale (CD-RISC);
5. Social Engagement (SE): 4 items adapted from the Chinese Longitudinal Healthy Longevity Survey (CLHLS) assessing social and family participation.

All items used 5-point Likert response options and were coded so that higher scores indicated better health status. Domain scores were computed as the mean of their respective items and scaled to 4–20, and the total health status score ranged from 20 to 100.

| **Domain** | **Variable** | **Value/Unit** |
| --- | --- | --- |
| General Health Perception (GHP) | In general, would you say your health is. | ① Excellent ② Very good ③ Good ④ Fair ⑤ Poor |
|  | I am as healthy as anybody I know. | ① Definitely true ② Mostly true ③ Not sure  ④ Mostly false ⑤ Definitely false |
|  | I seem to get sick a little more easily than other people. |  |
| Physical functioning (PF) | Vigorous activities (running, cycling, ball games, etc.) | ① Yes, limited a lot ② Yes, limited quite a bit  ③ Yes, limited moderately ④ Yes, limited a little ⑤ No, not limited at all |
|  | Moderate activities (cycling at regular pace, mopping, calisthenics) |  |
|  | Lifting/carrying groceries (<5kg) |  |
|  | Climbing one flight of stairs |  |
|  | Climbing three flights of stairs |  |
|  | Bending, kneeling, stooping |  |
|  | Walking 1000 meters |  |
|  | Walking 500 meters |  |
|  | Bathing or dressing yourself, cooking, etc. |  |
| Psychological well-being (PWB) | Did you feel full of life? | ① All of the time ② Most of the time ③ Some of the time ④ A little of the time ⑤ None of the time |
|  | Have you been a happy person? |  |
|  | Have you felt downhearted and blue? |  |
|  | Did you feel worn out? |  |
|  | Have you been a very nervous person? |  |
|  | Have you felt so down in the dumps that nothing could cheer you up? |  |
| Resilience (RES) | I am able to adapt to change. | ① Never ② Rarely ③ Sometimes  ④ Often ⑤ Always |
|  | I can deal with whatever comes my way. |  |
|  | I tend to bounce back after illness or hardship. |  |
|  | When things look hopeless, I don’t give up. |  |
|  | I know where to turn for help. |  |
|  | I am able to handle unpleasant feelings. |  |
| Social engagement (SE) | Participation in organized social activities | ① Almost daily ② Not daily, but ≥ once/week  ③ Not weekly, but ≥ once/month ④ Not monthly, but occasionally ⑤ Never |
|  | Participation in outdoor activities |  |
|  | Participation in family activities |  |
|  | How many times have you traveled away from home in the past two years? | ① 0 times ② 1 time ③ 2 times  ④ 3–4 times ⑤ ≥5 times |

1. **Questionnaire for psychological assessment**

| **Affect type** | **Questions** | Value/Unit |
| --- | --- | --- |
| Positive | You feel that your life is rich and fulfilling. How often does this describe your situation? | ① All of the time ② Most of the time ③ Some of the time ④ A little of the time ⑤ None of the time |
| Positive | Your mood is calm and peaceful. How often does this describe your situation? |  |
| Positive | Your mood is energetic and lively. How often does this describe your situation? |  |
| Positive | You are a happy person. How often does this describe your situation? |  |
| Positive | You feel as healthy as people around you. How often does this describe your situation? |  |
| Negative | Your mood is low. How often does this describe your situation? |  |
| Negative | You feel tired or exhausted. How often does this describe your situation? |  |
| Negative | You feel physically unwell. How often does this describe your situation? |  |
| Negative | Something is weighing heavily on your mind and you cannot shake it off. How often does this describe your situation? |  |
| Negative | You feel that your health is worse than that of other people. How often does this describe your situation? |  |

All items used 5-point Likert response options and were coded so that higher scores indicated more frequent occurrence of the positive/negative feeling, where 1 = None of the time and 5 = All of the time.

1. **Statistical analyses**

**Identify engagement trajectories**

To capture the longitudinal patterns of engagement, we applied latent class mixed models (LCMM) to participants’ weekly mobile health app recording frequencies using the *lcmm* package in R. LCMMs estimate unobserved subgroups that exhibit distinct temporal trajectories by modeling individual-level repeated measures. We fitted models with two to five latent classes and compared the Bayesian Information Criterion (BIC) for model selection. For each candidate model, we iteratively evaluated the functional form of time by fitting cubic, quadratic, and linear trajectory shapes in sequence. The optimal number of latent groups was selected based on (1) Bayesian Information Criterion (BIC), (2) average posterior probability >0.7, and (3) each group comprising at least 5 % of the total population. The final model supported a four-class solution, characterized by low-stable, increasing, decreasing, and high-stable engagement trajectories. Class membership was assigned using the maximum posterior probability rule for use in subsequent analyses.

**
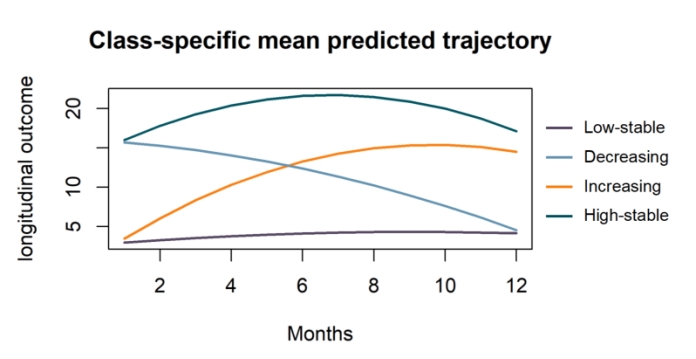
**

**Figure. Trajectories of weekly mobile health app usage frequency. The groups are labeled according to the initial value and the following trend.**

**Inverse probability of censoring weighting (IPCW)**

To account for potential informative dropout, we applied inverse probability of censoring weighting (IPCW). For each participant, we defined a binary indicator of observation at 12 months (1 = outcome observed; 0 = censored). We then fitted a logistic regression model for the probability of being observed at 12 months, using engagement groups, age, and sex as predictors. The predicted probability of being observed from this model was used to compute stabilized weights:

$$\text{SW}_{i}=\frac{P(R=1)}{\hat{P}(R_{i}=1\mid X_{i})},$$

where $P(R=1)$ is the marginal probability of observation and $\hat{P}(R_{i}=1\mid X_{i})$is the individual predicted probability from the censoring model. These stabilized weights were then applied in weighted linear mixed models estimating the association between engagement and changes in blood pressure, blood glucose, BMI and fat percentage outcomes.

**Mediation analysis**

**
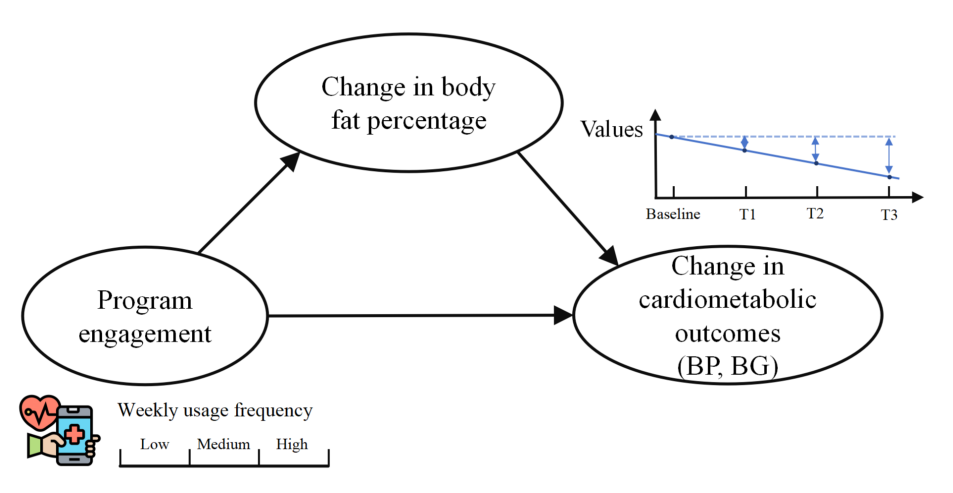
**

Figure. Program engagement is directly and indirectly (via change in body fat percentage) associated with change in cardiometabolic outcomes. Engagement was primarily measured by weekly mobile health app usage frequency; T1, T2, T3 represent timepoints.

To explore whether changes in body composition may partly account for the association between program engagement and cardiometabolic outcomes (blood pressure and blood glucose), we conducted exploratory mediation analyses. We modelled the mediator (change in body-fat percentage) and outcome (change in each cardiometabolic metric) using two mixed-effects models adjusted for age, sex, BMI, marital status, education, region, season, diabetes and hypertension status. Both models use all available repeated measurements. Mediation was then estimated with the *mediation* R package. Because the data are observational and timing of repeated measures is variable, these mediation analyses are generally exploratory.
