# Supplementary material for: A digital-health multidomain lifestyle management framework and its associations with cardiometabolic health: a real-world observational study
Source: BMC Med. 2026 Mar 25;24:285. doi: 10.1186/s12916-026-04830-y (PMC13137710; doi:10.1186/s12916-026-04830-y)
Supplement: Supplementary file 2 — Additional file 2: Table S1. The number of total health recordings and participants in each of the eight main datasets in the program; Table S2. Persistent use for the first year in the program; Table S3. Physiological, psychological, and lifestyle data by age and gender group at baseline; Table S4. Wearable use of participants across age and sex group; Table S5. Blood glucose, blood pressure, and body fat rate reduction over time by initial health status at baseline; Table S6. Associations Between Cardiometabolic Health and the Interactions of Baseline Status, Engagement, and Time Since Enrollment; Table S7. Adjusted Associations Between Blood Pressure, Blood Glucose, Body Composition, Engagement trajectories, and Time Since Enrollment; Table S8. Adjusted Associations Between Blood Pressure, Blood Glucose, Body Composition, Offline Participation, and Time Since Enrollment; Table S9. Adjusted Associations Between Blood Pressure, Blood Glucose, Body Composition, Engagement, and Time Since Enrollment, after inverse-probability-of-censoring weighted; Table S10. Adjusted Associations Between Blood Pressure, Blood Glucose, Body Composition, Engagement, and Time Since Enrollment, restricting to participants with over one year follow-up; Table S11. Mediating analysis of program engagement on BP, and BG through the change in body fat percentage; Figure S1. Flow chart of the study design; Figure S2. BG, BP, and body fat percentage change over 12-month follow-up with each month having at least four measurements [file 12916_2026_4830_MOESM2_ESM.docx]

Table S1. The number of total health recordings and participants in each of the eight main datasets in the program

| Health indicator | Total measurement amount | Number of participants | Average measurement amount |
| --- | --- | --- | --- |
| Blood pressure | 2,619,725 | 33,755 | 78 (238) |
| Blood glucose | 291,240 | 19,777 | 15 (54) |
| Body composition | 2,925,903 | 36,138 | 52 (181) |
| Heart rate | 8,623,004 | 26,682 | 323 (455) |
| Total cholesterol | 23,799 | 6,179 | 4 (11) |
| Hand grip strength | 83,923 | 13,378 | 6 (11) |
| Physical activity | 9,749,898 | 29,030 | 336 (458) |
| Sleep | 8,323,161 | 26,106 | 319 (449) |
| Mental health | 15,090 | 6,091 | 2.5 (1.5) |

Note: “Total measurement amount” is the total number of uploaded measurements in the dataset; “Number of participants” is defined as the number of unique users with ≥1 valid record in the dataset; “Average measurement amount” is the average number of recordings per person calculated as the ratio of total measurement amount and number of participants.

Table S2. Persistent use for the first year in the program.

| **Digital health program usage** | **≥ 1 month** | **≥ 6 month** | **≥ 12 month** |
| --- | --- | --- | --- |
| **Retention percentage for participants** |  |  |  |
| Blood pressure | 89% | 67% | 55% |
| Body composition | 88% | 64% | 52% |
| Blood glucose | 82% | 60% | 46% |
| Heart rate | 83% | 57% | 39% |
| Physical activity | 90% | 71% | 59% |
| Sleep | 91% | 75% | 62% |
| **Retention percentage for measurements** | **≥ 1 month** | **≥ 6 month** | **≥ 12 month** |
| Blood pressure | 96% | 84% | 72% |
| Body composition | 95% | 84% | 73% |
| Blood glucose | 82% | 60% | 45% |
| Heart rate | 95% | 79% | 67% |
| Physical activity | 96% | 80% | 64% |
| Sleep | 96% | 80% | 64% |

Table S3. Physiological, psychological, and lifestyle data by age and gender group at baseline.

|  | **18-40 years** | |  | **40-65 years** | |  | **65-75 years** | |  | **≥75 years** |  |
| --- | --- | --- | --- | --- | --- | --- | --- | --- | --- | --- | --- |
|  | Male | Female |  | Male | Female |  | Male | Female |  | Male | Female |
| **Physiological parameters** | | | | | | | | | | | |
| **Body composition indices (n = 18027)** | **(n = 429)** | **(n = 884)** |  | **(n = 829)** | **(n = 2695)** |  | **(n = 2654)** | **(n = 5649)** |  | **(n = 2000)** | **(n = 2887)** |
| Body mass index, kg/m2 | 24.8 (4.1) | 23.7 (4.2) |  | 25.1 (3.5) | 24.8 (3.5) |  | 24.8 (3.2) | 24.6 (3.5) |  | 24.1 (3.2) | 24.1 (3.4) |
| Body fat percentage, % | 24.5 (8.5) | 30.3 (6.2) |  | 25.6 (5.9) | 34.8 (5.2) |  | 25.4 (6) | 35.2 (5.8) |  | 24.8 (6.7) | 34.9 (6.1) |
| Body water percentage, % | 53.3 (5.3) | 48.2 (4.1) |  | 53.1 (4) | 46.1 (3.5) |  | 53.4 (3.9) | 46.2 (4.2) |  | 54 (4.3) | 46.7 (4.6) |
| Bone weight, kg | 2.8 (0.4) | 2.5 (0.4) |  | 2.7 (0.3) | 2.4 (0.3) |  | 2.7 (0.3) | 2.3 (0.3) |  | 2.6 (0.3) | 2.2 (0.3) |
| **Blood pressure indices (n = 14539)** | **(n = 216)** | **(n = 409)** |  | **(n = 622)** | **(n = 1991)** |  | **(n = 2206)** | **(n = 4819)** |  | **(n = 1696)** | **(n = 2580)** |
| Systolic blood pressure, mm Hg | 123.6 (15.4) | 112.5 (17.1) |  | 127.6 (16.4) | 124.3 (16.6) |  | 129.3 (16.7) | 127.9 (16.6) |  | 131.2 (16.7) | 130.5 (16.5) |
| Diastolic blood pressure, mm Hg | 75.1 (11.2) | 68.9 (11.5) |  | 78.6 (11.3) | 74.9 (11.3) |  | 75.2 (10.6) | 72.1 (10.8) |  | 72.5 (10.4) | 70.3 (10.5) |
| Mean arterial pressure, mm Hg | 91.2 (11.3) | 83.4 (12.2) |  | 94.9 (11.9) | 91.4 (11.8) |  | 93.2 (11.2) | 90.7 (11.2) |  | 92 (10.8) | 90.4 (10.9) |
| Pulse pressure, mm Hg | 48.5 (12.3) | 43.6 (12.6) |  | 49 (12.4) | 49.4 (12.8) |  | 54.1 (14.1) | 55.7 (14) |  | 58.7 (14.7) | 60.2 (14.3) |
| **Heart rate indicies (n = 12833)** | **(n = 356)** | **(n = 438)** |  | **(n = 651)** | **(n = 1775)** |  | **(n = 1884)** | **(n = 4035)** |  | **(n = 1490)** | **(n = 2204)** |
| Daily average heart rate, counts/min | 79 (8.2) | 78.7 (8.5) |  | 76.4 (8) | 75 (7.5) |  | 74.3 (7.6) | 74 (7.1) |  | 72.7 (7.1) | 73.4 (6.7) |
| Daily standard deviation of heart rate, counts/min | 12.6 (4.5) | 12.6 (4.3) |  | 11.8 (4.1) | 11.2 (3.9) |  | 10.8 (3.8) | 10.6 (3.9) |  | 10.1 (3.7) | 9.8 (3.7) |
| **Glycemic control indices (n = 7523)** | **(n = 69)** | **(n = 140)** |  | **(n = 323)** | **(n = 1163)** |  | **(n = 1203)** | **(n = 2693)** |  | **(n = 747)** | **(n = 1185)** |
| Daily blood glucose, mmol/L | 7 (2.5) | 6.6 (2.4) |  | 7.9 (2.4) | 7.6 (2.5) |  | 7.8 (2.5) | 7.7 (2.5) |  | 7.8 (2.6) | 7.7 (2.5) |
| **Muscle strength indices (n = 6943)** | **(n = 331)** | **(n = 606)** |  | **(n = 567)** | **(n = 1740)** |  | **(n = 1961)** | **(n = 4334)** |  | **(n = 1463)** | **(n = 2165)** |
| Hand grip strength, kg | 42.9 (10.2) | 36.8 (8.6) |  | 40 (7.4) | 32.1 (8.8) |  | 38.8 (7) | 30.9 (9.2) |  | 36.1 (7.8) | 28.4 (9.6) |
| **Blood lipids indices (n = 6128)** | **(n = 63)** | **(n = 106)** |  | **(n = 263)** | **(n = 824)** |  | **(n = 954)** | **(n = 2028)** |  | **(n = 751)** | **(n = 1139)** |
| Daily total cholesterol, mmol/L | 6.1 (2.1) | 4.8 (1.6) |  | 5.7 (1.9) | 6 (1.7) |  | 5.9 (1.8) | 5.7 (1.8) |  | 5.9 (1.8) | 6.1 (1.7) |
| **Lifestyle parameters** | | | | | | | | | | | |
| **Sleep parameters (n = 14531)** | **(n = 219)** | **(n = 350)** |  | **(n = 607)** | **(n = 1849)** |  | **(n = 1884)** | **(n = 4269)** |  | **(n = 1559)** | **(n = 2375)** |
| Total sleep duration, min | 402 (130.7) | 411.4 (116.7) |  | 409.3 (113.9) | 412.3 (106.3) |  | 418.7 (112.6) | 416.5 (107.5) |  | 428.5 (114) | 421.5 (108.9) |
| Deep sleep duration, min | 102.3 (70.4) | 108.4 (66.5) |  | 90.6 (59.6) | 112.7 (55.1) |  | 102 (58.1) | 119.9 (56.9) |  | 108.7 (58.6) | 125.3 (56.5) |
| Deep sleep duration/total sleep duration, % | 24.9 (14.5) | 26.1 (13.7) |  | 21.8 (12.5) | 27.4 (11.6) |  | 24.1 (11.8) | 28.8 (11.6) |  | 25.1 (11.7) | 29.7 (11.4) |
| Sleep regularity index | 91.2 (8.1) | 92.1 (7.8) |  | 91.8 (7.3) | 91.8 (7.2) |  | 91.8 (7.5) | 91.7 (7.5) |  | 92.1 (7.4) | 91.7 (7.6) |
| **Physical activity parameters (n = 15738)** | **(n = 373)** | **(n = 486)** |  | **(n = 781)** | **(n = 2211)** |  | **(n = 2297)** | **(n = 5009)** |  | **(n = 1831)** | **(n = 2750)** |
| Daily step counts, steps | 5802.5 (4204.7) | 5544.6 (3850.9) |  | 7215.3 (4934) | 7364.2 (4905.5) |  | 7469.3 (4956.6) | 7347.3 (4790.1) |  | 6998.4 (4683.1) | 6701.2 (4367.4) |
| Daily walking or running distance, meters | 4185.2 (3059.9) | 3861.5 (2726) |  | 5190 (3627.4) | 5087.8 (3469.6) |  | 5363.6 (3612.6) | 5108.2 (3437.5) |  | 5005.2 (3405.6) | 4640.9 (3137.3) |
| **Psychological parameters** | | | | | | | | | | | |
| **Self-designed questionnaire (n = 6085)** | **(n = 75)** | **(n = 170)** |  | **(n = 418)** | **(n = 1278)** |  | **(n = 999)** | **(n = 2216)** |  | **(n = 316)** | **(n = 613)** |
| Positive emotions | 17.5 (4.8) | 18.1 (4.3) |  | 18.2 (4.8) | 18.7 (4.3) |  | 18.9 (4.1) | 18.8 (4.1) |  | 19.3 (3.7) | 19 (4.1) |
| Negative emotions | 12.1 (5.4) | 11.2 (3.9) |  | 9.1 (3.8) | 9.4 (3.6) |  | 9.2 (3.7) | 9.6 (3.6) |  | 9.2 (3.7) | 9.4 (3.5) |

Data are displayed from subjects who have ≥ 2 measurements in the first month. Continuous variables are presented as mean (SD).

Table S4. Wearable use of participants across age and sex group.

|  | **18-40 years** |  |  | **40-65 years** |  |  | **65-75 years** |  |  | **≥**75 years |  |
| --- | --- | --- | --- | --- | --- | --- | --- | --- | --- | --- | --- |
|  | Male | Female |  | Male | Female |  | Male | Female |  | Male | Female |
| Total time of worn (days) | 115.9 (201.4)^a^ | 157.6 (253.6) |  | 317.3 (401.1) | 406.4 (452.1) |  | 481.2 (525.6) | 516.4 (547.6) |  | 473.9 (549.7) | 531.6 (586.6) |
| Number of days per week worn | 4 (1.5) | 4.2 (1.4) |  | 4.9 (1.6) | 5.1 (1.4) |  | 5.3 (1.4) | 5.3 (1.3) |  | 5.2 (1.4) | 5.1 (1.4) |
| Daily wear time (hours) | 11.7 (4.4) | 13.1 (4.5) |  | 15.0 (4.9) | 16.1 (4.6) |  | 16.2 (4.6) | 16.6 (4.5) |  | 17.0 (4.4) | 17.2 (4.4) |

Continuous variables are presented as mean (SD).

Table S5. Blood glucose, blood pressure, and body fat rate reduction over time by initial health status at baseline

| Time from enrollment | Induced months | Enrolled participants^a^ | | | Participants who reduced cardiometabolic metrics compared to baseline, No. (%) | | | Mean difference in cardiometabolic and lifestyles metrics (SEM)^b^ | | | | |
| --- | --- | --- | --- | --- | --- | --- | --- | --- | --- | --- | --- | --- |
|  |  | SBP | BG | Body fat percentage | SBP | BG | Body fat percentage | SBP | BG | Body fat percentage | Daily steps | Sleep regularity index |
| Participants with elevated BG | | | | | | | | | | | | |
| 2 month | 2 month | 920 | 600 | 1030 | 500 (54%) | 463 (77%) | 567 (58%) | -9.1 (0.4) | -2.4 (0.1) | -1.15 (0.12) | 1765.7 (79.9) | 5.3 (0.2) |
| 4 month | 3-4 month | 952 | 610 | 1062 | 521 (55%) | 491 (80%) | 596 (60%) | -10.2 (0.4) | -2.9 (0.1) | -1.61 (0.14) | 2087.2 (94.6) | 5.3 (0.2) |
| 6 month | 5-6 month | 830 | 475 | 913 | 450 (54%) | 395 (83%) | 500 (60%) | -11.2 (0.5) | -3 (0.1) | -2.06 (0.16) | 2288.9 (109.5) | 5 (0.2) |
| 12 month | 10-12 month | 733 | 323 | 793 | 392 (53%) | 273 (85%) | 360 (58%) | -11.4 (0.5) | -3.5 (0.2) | -2.23 (0.2) | 2714.1 (141.6) | 5.4 (0.2) |
| Participants with elevated BP | | | | | | | | | | | | |
| 2 month | 2 month | 2090 | 848 | 2446 | 1558 (75%) | 513 (61%) | 1400 (57%) | -12.9 (0.27) | -1.24 (0.06) | -1.19 (0.07) | 1985.4 (56.8) | 5 (0.1) |
| 4 month | 3-4 month | 2276 | 884 | 2605 | 1814 (80%) | 553 (59%) | 1499 (58%) | -14.83 (0.27) | -1.31 (0.06) | -1.54 (0.08) | 2187.9 (66.2) | 5.5 (0.1) |
| 6 month | 5-6 month | 1961 | 813 | 2195 | 1571 (80%) | 489 (60%) | 1290 (59%) | -16.39 (0.31) | -1.46 (0.07) | -1.96 (0.09) | 2445.1 (76.4) | 5.3 (0.1) |
| 12 month | 10-12 month | 1543 | 482 | 1833 | 1253 (81%) | 274 (57%) | 826 (45%) | -17.03 (0.36) | -1.55 (0.11) | -2.18 (0.12) | 2718.4 (97.2) | 5.4 (0.1) |
| Participants with overweight | | | | | | | | | | | | |
| 2 month | 2 month | 4213 | 1731 | 6730 | 2364 (56%) | 1017 (59%) | 4741 (73%) | -8.62 (0.17) | -1.13 (0.04) | -1.04 (0.02) | 1865 (37.7) | 4.9 (0.1) |
| 4 month | 3-4 month | 4358 | 1933 | 6678 | 2502 (57%) | 1136 (59%) | 4885 (76%) | -9.72 (0.18) | -1.23 (0.04) | -1.42 (0.03) | 2226.2 (46.1) | 5.5 (0.1) |
| 6 month | 5-6 month | 3710 | 1558 | 5356 | 2067 (56%) | 943 (61%) | 3836 (72%) | -10.98 (0.21) | -1.28 (0.04) | -1.86 (0.04) | 2535.4 (54.4) | 5.3 (0.1) |
| 12 month | 10-12 month | 2843 | 1006 | 3843 | 1599 (56%) | 605 (60%) | 2346 (61%) | -10.68 (0.24) | -1.53 (0.07) | -2.42 (0.05) | 2902.1 (70.5) | 5.6 (0.1) |

BG, blood glucose; SBP, systolic blood pressure; SEM, standard error of mean. Elevated BG was defined as a BG of 10 mmol/L or higher; Elevated BP was defined as either systolic BP ≥ 140 mmHg or diastolic BP ≥ 90 mmHg; Overweight was defined as body fat rate ≥ 25% for males and ≥ 36% for females. ^a^Number of participants at each time represents participants who had been enrolled in the programlong enough to have reached that point and who had application activity during the induced months listed. Sinceparticipants enrolled in the program at different times during the study period, duration of follow-up was not the same for all participants and not all participants provided data for all time points. ^b^Differences were calculated only in participants who reduced SBP, BG, or body fat percentage.

Table S6.Associations Between Cardiometabolic Health and the Interactions of Baseline Status, Engagement, and Time Since Enrollment

| **Health indicators** | **Estimate (95%CI)** | **P value** |
| --- | --- | --- |
| **Change in Systolic Blood Pressure^a^** |  |  |
| Elevated BP:Time change | 10.47 (9.12, 11.81) | <0.001 |
| Elevated BP:Medium engagement | -1.89 (-3.27, -0.51) | 0.007 |
| Elevated BP:High engagement | -3.47 (-4.79, -2.15) | <0.001 |
| Elevated BP:Medium engagement:Time change | 2.03 (0.56, 3.5) | 0.007 |
| Elevated BP:High engagement:Time change | 5.34 (3.95, 6.74) | <0.001 |
| **Change in Diastolic Blood Pressure** |  |  |
| Elevated BP:Time change | 4.32 (3.44, 5.2) | <0.001 |
| Elevated BP:Medium engagement | -0.39 (-1.31, 0.54) | 0.412 |
| Elevated BP:High engagement | -0.53 (-1.41, 0.36) | 0.241 |
| Elevated BP:Medium engagement:Time change | 0.57 (-0.4, 1.53) | 0.249 |
| Elevated BP:High engagement:Time change | 1.46 (0.54, 2.38) | 0.002 |
| **Change in Blood Glucose^b^** |  |  |
| Elevated BG:Time change | 2.05 (1.38, 2.73) | <0.001 |
| Elevated BG:Medium engagement | -0.06 (-0.7, 0.59) | 0.863 |
| Elevated BG:High engagement | -1.21 (-1.85, -0.58) | <0.001 |
| Elevated BG:Medium engagement:Time change | 0.23 (-0.47, 0.93) | 0.524 |
| Elevated BG:High engagement:Time change | 1.25 (0.57, 1.94) | <0.001 |
| **Change in Body fat rate^c^** |  |  |
| Overweight:Time change | 1.07 (0.83, 1.31) | <0.001 |
| Obesity:Time change | 1.87 (1.49, 2.26) | <0.001 |
| Overweight:Medium engagement | -0.95 (-1.24, -0.66) | <0.001 |
| Overweight:High engagement | -2.5 (-2.78, -2.23) | <0.001 |
| Obesity:Medium engagement | -1.33 (-1.79, -0.87) | <0.001 |
| Obesity:High engagement | -3.21 (-3.65, -2.76) | <0.001 |
| Overweight:Medium engagement:Time change | 1.03 (0.75, 1.31) | <0.001 |
| Overweight:High engagement:Time change | 2.5 (2.24, 2.76) | <0.001 |
| Obesity:Medium engagement:Time change | 1.53 (1.09, 1.98) | <0.001 |
| Obesity:High engagement:Time change | 3.62 (3.19, 4.04) | <0.001 |
| **Change in BMI^d^** |  |  |
| Normal weight:Time change | 0.42 (0.21, 0.64) | <0.001 |
| Overweight:Time change | 0.77 (0.55, 1) | <0.001 |
| Obesity:Time change | 1.24 (0.95, 1.52) | <0.001 |
| Normal weight:Medium engagement | -0.45 (-0.73, -0.18) | 0.001 |
| Normal weight:High engagement | -0.24 (-0.51, 0.03) | 0.084 |
| Overweight:Medium engagement | -0.82 (-1.1, -0.54) | <0.001 |
| Overweight:High engagement | -1.34 (-1.62, -1.06) | <0.001 |
| Obesity:Medium engagement | -0.94 (-1.3, -0.58) | <0.001 |
| Obesity:High engagement | -1.46 (-1.81, -1.11) | <0.001 |
| Normal weight:Medium engagement:Time change | 0.56 (0.3, 0.81) | <0.001 |
| Normal weight:High engagement:Time change | 0.82 (0.57, 1.06) | <0.001 |
| Overweight:Medium engagement:Time change | 0.94 (0.69, 1.2) | <0.001 |
| Overweight:High engagement:Time change | 1.91 (1.66, 2.16) | <0.001 |
| Obesity:Medium engagement:Time change | 1.29 (0.96, 1.62) | <0.001 |
| Obesity:High engagement:Time change | 2.27 (1.95, 2.59) | <0.001 |

^a^Elevated BP was defined as either systolic BP ≥ 140 mmHg or diastolic BP ≥ 90 mmHg; ^b^ Elevated BG was defined as a BG of 10 mmol/L or higher; ^c^ Obesity was defined as body fat rate ≥ 30% for males and ≥ 42% for females; Overweight was defined as body fat rate ≥ 25% for males and ≥ 36% for females. ^d^Obesity was defined as BMI ≥ 30 kg/m^2^; Overweight was defined as 30 kg/m^2^ ≥ BMI > 25 kg/m^2^; Normal weight was defined as 25 kg/m^2^ ≥ BMI > 18.5 kg/m^2^.

Table S7. Adjusted Associations Between Blood Pressure, Blood Glucose, Body Composition, Engagement trajectories, and Time Since Enrollment

| **Health indicators** | **Time change^e^** | **Decreasing (vs. Low-stable) engagement** | **Increasing (vs. Low-stable) engagement** | **High-stable (vs. Low-stable) engagement** | **Decreasing × Time change** | **Increasing × Time change** | **High-stable × Time change** |
| --- | --- | --- | --- | --- | --- | --- | --- |
| **Change in SBP^a^** |  |  |  |  |  |  |  |
| Participants with elevated BP | 13.0 (12.6, 13.5)^f^ | -0.02 (-0.93, 0.88) | -1.14 (-2.04, -0.24) | -0.52 (-1.32, 0.28) | 2.75 (1.88, 3.61) | 2 (1.12, 2.89) | 4.26 (3.52, 5) |
| Participants with normal BP | -0.7 (-0.94, -0.5) | -0.7 (-1.07, -0.32) | 0.02 (-0.34, 0.38) | -1.61 (-1.93, -1.28) | 1.67 (1.26, 2.07) | 0.01 (-0.39, 0.4) | 2.53 (2.19, 2.86) |
| **Change in DBP** |  |  |  |  |  |  |  |
| Participants with elevated BP | 5.25 (4.96, 5.54) | 0.34 (-0.25, 0.93) | 0.08 (-0.51, 0.67) | -0.37 (-0.89, 0.16) | 0.47 (-0.06, 1) | -0.12 (-0.66, 0.42) | 1.64 (1.18, 2.09) |
| Participants with normal BP | -0.14 (-0.29, 0) | -0.19 (-0.45, 0.07) | -0.35 (-0.6, -0.09) | -0.79 (-1.01, -0.56) | 0.49 (0.22, 0.76) | 0.29 (0.03, 0.55) | 0.94 (0.72, 1.17) |
| **Change in BG^b^** |  |  |  |  |  |  |  |
| Participants with elevated BG | 2.77 (2.57, 2.96) | -0.29 (-0.61, 0.03) | -0.54 (-0.84, -0.24) | -0.91 (-1.2, -0.63) | 0.21 (-0.13, 0.54) | 0.43 (0.1, 0.77) | 1.18 (0.88, 1.48) |
| Participants with normal BG | -0.01 (-0.06, 0.04) | -0.09 (-0.16, -0.03) | -0.06 (-0.12, 0) | -0.15 (-0.2, -0.09) | 0.26 (0.18, 0.34) | 0.14 (0.05, 0.22) | 0.29 (0.22, 0.36) |
| **Change in Body fat percentage^c^** |  |  |  |  |  |  |  |
| Participants with obesity | 1.62 (1.47, 1.77) | -0.42 (-0.74, -0.09) | -0.79 (-1.18, -0.41) | -0.69 (-1.01, -0.38) | 0.73 (0.42, 1.04) | 1.85 (1.47, 2.23) | 1.35 (1.06, 1.65) |
| Participants with overweight | 0.36 (0.3, 0.43) | -0.24 (-0.34, -0.13) | -0.32 (-0.44, -0.2) | -0.55 (-0.64, -0.45) | 0.36 (0.21, 0.5) | 0.42 (0.25, 0.58) | 0.92 (0.79, 1.05) |
| Participants with healthy weight | -2.26 (-2.33, -2.18) | -0.08 (-0.3, 0.15) | 1.74 (1.54, 1.94) | -1.62 (-1.81, -1.43) | -0.31 (-0.48, -0.15) | -1.71 (-1.86, -1.55) | 1.18 (1.05, 1.31) |
| **Change in BMI^d^** |  |  |  |  |  |  |  |
| Participants with obesity | 1.39 (1.3, 1.49) | 0.03 (-0.2, 0.27) | -0.22 (-0.48, 0.04) | -0.24 (-0.46, -0.03) | 0.28 (0.06, 0.49) | 0.04 (-0.2, 0.28) | 0.76 (0.57, 0.94) |
| Participants with overweight | 0.57 (0.54, 0.6) | -0.11 (-0.17, -0.05) | -0.23 (-0.3, -0.16) | -0.42 (-0.48, -0.37) | 0.28 (0.22, 0.35) | 0.37 (0.3, 0.45) | 0.78 (0.72, 0.84) |
| Participants with normal weight | -0.32 (-0.35, -0.3) | 0.02 (-0.06, 0.1) | 0.71 (0.64, 0.79) | -0.56 (-0.62, -0.49) | 0.01 (-0.04, 0.06) | -0.6 (-0.65, -0.55) | 0.6 (0.55, 0.64) |
| Participants with underweight | -1.09 (-1.21, -0.97) | 0.24 (-0.13, 0.62) | 0.03 (-0.37, 0.42) | 0.22 (-0.1, 0.54) | -0.89 (-1.17, -0.61) | -0.16 (-0.48, 0.16) | -0.35 (-0.58, -0.11) |

Engagement trajectories were identified using latent class mixed model. ^a^ Elevated BP was defined as either systolic BP ≥ 140 mmHg or diastolic BP ≥ 90 mmHg; ^b^ Elevated BG was defined as a BG of 10 mmol/L or higher; ^c^ Obesity was defined as body fat rate ≥ 30% for males and ≥ 42% for females; Overweight was defined as body fat rate ≥ 25% for males and ≥ 36% for females. ^d^Obesity was defined as BMI ≥ 30 kg/m^2^; Overweight was defined as 30 kg/m^2^ ≥ BMI > 25 kg/m^2^; Normal weight was defined as 25 kg/m^2^ ≥ BMI > 18.5 kg/m^2^. ^e^Time was transformed with a negative exponent to account for the non-linear time effect, calculated as exp(-time since start in weeks). A positive coefficient represents a decreasing trend. ^f^parenthetical values represent 95% confidence intervals

Table S8. Adjusted Associations Between Blood Pressure, Blood Glucose, Body Composition, Offline Participation, and Time Since Enrollment

| **Health indicators** | **Unweighted** | | |  | **IPCW weighted^g^** | | |
| --- | --- | --- | --- | --- | --- | --- | --- |
|  | **Time change^e^** | **Offline activate (vs. inactive)** | **Offline activate × Time change** |  | **Time change** | **Offline activate (vs. inactive)** | **Offline activate × Time change** |
| **Change in SBP^a^** |  |  |  |  |  |  |  |
| Participants with elevated BP | 12.83 (12.3, 13.35)^f^ | -1.15 (-1.8, -0.49) | 3.12 (2.48, 3.76) |  | 15.06 (13.97, 16.15) | -1.92 (-3.24, -0.59) | 2.45 (1.29, 3.62) |
| Participants with normal BP | -1.28 (-1.53, -1.03) | -1.2 (-1.48, -0.92) | 2.18 (1.88, 2.48) |  | -1.82 (-2.37, -1.26) | -1.67 (-2.25, -1.09) | 2.22 (1.64, 2.81) |
| **Change in DBP** |  |  |  |  |  |  |  |
| Participants with elevated BP | 4.61 (4.28, 4.93) | -0.73 (-1.16, -0.31) | 1.66 (1.27, 2.05) |  | 4.91 (4.24, 5.57) | -0.95 (-1.87, -0.03) | 1.54 (0.83, 2.25) |
| Participants with normal BP | -0.46 (-0.63, -0.29) | -0.67 (-0.87, -0.47) | 1 (0.8, 1.2) |  | -0.76 (-1.13, -0.39) | -1.02 (-1.45, -0.59) | 1.1 (0.71, 1.49) |
| **Change in BG^b^** |  |  |  |  |  |  |  |
| Participants with elevated BG | 3.14 (2.91, 3.37) | -0.25 (-0.48, -0.02) | 0.13 (-0.14, 0.39) |  | 3.71 (3.29, 4.13) | -0.54 (-0.93, -0.16) | 0.05 (-0.4, 0.5) |
| Participants with normal BG | 0.05 (-0.01, 0.11) | -0.02 (-0.07, 0.04) | 0.16 (0.09, 0.23) |  | -0.21 (-0.34, -0.08) | -0.14 (-0.23, -0.05) | 0.28 (0.14, 0.42) |
| **Change in Body fat percentage^c^** |  |  |  |  |  |  |  |
| Participants with obesity | 1.89 (1.72, 2.06) | -0.11 (-0.35, 0.12) | 0.6 (0.38, 0.83) |  | 2.84 (2.44, 3.25) | 0.03 (-0.67, 0.73) | 0.7 (0.26, 1.15) |
| Participants with overweight | 0.77 (0.7, 0.85) | -0.16 (-0.24, -0.08) | -0.11 (-0.21, -0.01) |  | 1.06 (0.86, 1.25) | 0.09 (-0.12, 0.31) | -0.22 (-0.43, -0.01) |
| Participants with healthy weight | -1.72 (-1.8, -1.65) | 0.67 (0.53, 0.82) | -0.93 (-1.04, -0.83) |  | -2.61 (-2.78, -2.44) | 0.1 (-0.24, 0.43) | -0.61 (-0.8, -0.42) |
| **Change in BMI^d^** |  |  |  |  |  |  |  |
| Participants with obesity | 1.52 (1.41, 1.63) | 0.12 (-0.04, 0.28) | 0.2 (0.05, 0.34) |  | 1.91 (1.65, 2.17) | 0.07 (-0.37, 0.51) | 0.18 (-0.11, 0.46) |
| Participants with overweight | 0.58 (0.55, 0.62) | -0.26 (-0.3, -0.22) | 0.47 (0.42, 0.51) |  | 0.7 (0.62, 0.77) | -0.35 (-0.45, -0.24) | 0.45 (0.37, 0.54) |
| Participants with normal weight | -0.44 (-0.47, -0.41) | -0.13 (-0.18, -0.09) | 0.28 (0.24, 0.31) |  | -0.82 (-0.88, -0.77) | -0.37 (-0.49, -0.25) | 0.43 (0.36, 0.49) |
| Participants with underweight | -1.17 (-1.31, -1.04) | 0.03 (-0.19, 0.26) | -0.26 (-0.44, -0.08) |  | -1.79 (-2.08, -1.5) | 0.01 (-0.57, 0.59) | 0.12 (-0.2, 0.43) |

^a^ Elevated BP was defined as either systolic BP ≥ 140 mmHg or diastolic BP ≥ 90 mmHg; ^b^ Elevated BG was defined as a BG of 10 mmol/L or higher; ^c^ Obesity was defined as body fat rate ≥ 30% for males and ≥ 42% for females; Overweight was defined as body fat rate ≥ 25% for males and ≥ 36% for females. ^d^Obesity was defined as BMI ≥ 30 kg/m^2^; Overweight was defined as 30 kg/m^2^ ≥ BMI > 25 kg/m^2^; Normal weight was defined as 25 kg/m^2^ ≥ BMI > 18.5 kg/m^2^. ^e^Time was transformed with a negative exponent to account for the non-linear time effect, calculated as exp(-time since start in weeks). A positive coefficient represents a decreasing trend. ^f^parenthetical values represent 95% confidence intervals.^g^ inverse-probability-of-censoring weighting was applied to address informative dropping out.

Table S9. Adjusted Associations Between Blood Pressure, Blood Glucose, Body Composition, Engagement, and Time Since Enrollment, after inverse-probability-of-censoring weighted (IPCW)

| **Health indicators** | **Time change^e^** | **Medium (vs. Low) engagement** | **High (vs. Low) engagement** | **Medium engagement × Time change** | **High engagement × Time change** |
| --- | --- | --- | --- | --- | --- |
| **Change in SBP^a^** |  |  |  |  |  |
| Participants with elevated BP | 10.7 (9.84, 11.56)^f^ | -2.83 (-5.47, -0.19) | -3.63 (-6.2, -1.06) | 3.5 (2.46, 4.54) | 6.49 (5.54, 7.45) |
| Participants with normal BP | -1.02 (-1.46, -0.58) | 0.57 (-0.71, 1.85) | -0.51 (-1.76, 0.74) | -0.36 (-0.88, 0.16) | 1.2 (0.72, 1.68) |
| **Change in DBP** |  |  |  |  |  |
| Participants with elevated BP | 4.11 (3.59, 4.64) | -1.37 (-3.21, 0.47) | -1.34 (-3.13, 0.44) | 1.92 (1.28, 2.55) | 2.33 (1.75, 2.91) |
| Participants with normal BP | 0.33 (0.03, 0.62) | 0.61 (-0.35, 1.58) | 0.24 (-0.7, 1.18) | -0.48 (-0.82, -0.13) | -0.15 (-0.46, 0.17) |
| **Change in BG^b^** |  |  |  |  |  |
| Participants with elevated BG | 2.67 (1.91, 3.43) | 0.84 (-1.17, 2.86) | -0.02 (-2.01, 1.97) | 0.13 (-0.66, 0.92) | 1.37 (0.59, 2.15) |
| Participants with normal BG | -0.37 (-0.51, -0.22) | 0.01 (-0.34, 0.35) | -0.16 (-0.51, 0.18) | 0.22 (0.06, 0.38) | 0.47 (0.31, 0.62) |
| **Change in Body fat percentage^c^** |  |  |  |  |  |
| Participants with obesity | 2.46 (2.22, 2.7) | 0.25 (-0.89, 1.39) | -1.10 (-2.13, -0.08) | 0.09 (-0.23, 0.41) | 1.21 (0.88, 1.53) |
| Participants with overweight | 0.35 (0.23, 0.47) | -0.02 (-0.35, 0.32) | -0.59 (-0.91, -0.28) | -0.32 (-0.48, -0.16) | 0.77 (0.62, 0.92) |
| Participants with healthy weight | -1.33 (-1.43, -1.22) | 1.26 (0.7, 1.82) | 1.58 (1.05, 2.11) | -1.14 (-1.28, -1) | -1.71 (-1.84, -1.58) |
| **Change in BMI^d^** |  |  |  |  |  |
| Participants with obesity | 1.53 (1.37, 1.7) | -0.02 (-0.74, 0.71) | -0.26 (-0.96, 0.45) | 0.48 (0.27, 0.7) | 0.51 (0.31, 0.72) |
| Participants with overweight | 0.58 (0.52, 0.63) | -0.05 (-0.25, 0.15) | -0.42 (-0.61, -0.23) | -0.06 (-0.13, 0.01) | 0.57 (0.5, 0.64) |
| Participants with normal weight | -0.08 (-0.11, -0.04) | 0.47 (0.25, 0.69) | 0.72 (0.51, 0.93) | -0.27 (-0.32, -0.23) | -0.45 (-0.5, -0.41) |
| Participants with underweight | -0.44 (-0.66, -0.22) | 0.75 (-0.37, 1.87) | 0.84 (-0.26, 1.93) | -1.51 (-1.8, -1.23) | -1.73 (-2.02, -1.44) |

^a^ Elevated BP was defined as either systolic BP ≥ 140 mmHg or diastolic BP ≥ 90 mmHg; ^b^ Elevated BG was defined as a BG of 10 mmol/L or higher; ^c^ Obesity was defined as body fat rate ≥ 30% for males and ≥ 42% for females; Overweight was defined as body fat rate ≥ 25% for males and ≥ 36% for females. ^d^Obesity was defined as BMI ≥ 30 kg/m^2^; Overweight was defined as 30 kg/m^2^ ≥ BMI > 25 kg/m^2^; Normal weight was defined as 25 kg/m^2^ ≥ BMI > 18.5 kg/m^2^. ^e^Time was transformed with a negative exponent to account for the non-linear time effect, calculated as exp(-time since start in weeks). A positive coefficient represents a decreasing trend. ^f^parenthetical values represent 95% confidence intervals

Table S10. Adjusted Associations Between Blood Pressure, Blood Glucose, Body Composition, Engagement, and Time Since Enrollment, restricting to participants with over one year follow-up

| **Health indicators** | **Time change^e^** | **Medium (vs. Low) engagement** | **High (vs. Low) engagement** | **Medium engagement × Time change** | **High engagement × Time change** |
| --- | --- | --- | --- | --- | --- |
| **Change in SBP^a^** |  |  |  |  |  |
| Participants with elevated BP | 7.54 (6.33, 8.74)^f^ | -1.94 (-3.4, -0.48) | -1.57 (-2.96, -0.18) | 2.8 (1.31, 4.3) | 2.07 (0.7, 3.44) |
| Participants with normal BP | -1.19 (-1.7, -0.67) | -0.08 (-0.61, 0.46) | -0.08 (-0.59, 0.43) | 0.38 (-0.23, 0.99) | 0.21 (-0.35, 0.78) |
| **Change in DBP** |  |  |  |  |  |
| Participants with elevated BP | 2.66 (1.94, 3.38) | -0.24 (-1.18, 0.7) | -0.31 (-1.2, 0.59) | 0.43 (-0.46, 1.32) | 0.45 (-0.36, 1.27) |
| Participants with normal BP | -0.48 (-0.83, -0.13) | 0.18 (-0.21, 0.57) | -0.43 (-0.8, -0.06) | -0.17 (-0.58, 0.24) | 0.47 (0.09, 0.85) |
| **Change in BG^b^** |  |  |  |  |  |
| Participants with elevated BG | 1.74 (1.1, 2.38) | -1.44 (-2.21, -0.66) | -1.21 (-1.94, -0.49) | 1.4 (0.61, 2.19) | 1.3 (0.57, 2.03) |
| Participants with normal BG | -0.19 (-0.35, -0.03) | 0.14 (-0.01, 0.29) | 0.04 (-0.1, 0.18) | -0.1 (-0.29, 0.09) | -0.01 (-0.19, 0.16) |
| **Change in Body fat percentage^c^** |  |  |  |  |  |
| Participants with obesity | 0.93 (0.6, 1.26) | -0.85 (-1.31, -0.39) | -0.53 (-1, -0.07) | 1.57 (1.12, 2.02) | 0.55 (0.1, 1) |
| Participants with overweight | -0.01 (-0.15, 0.14) | -0.28 (-0.43, -0.12) | -0.24 (-0.39, -0.1) | 0.54 (0.35, 0.73) | 0.31 (0.14, 0.49) |
| Participants with healthy weight | -1.19 (-1.35, -1.02) | -0.07 (-0.29, 0.16) | -0.26 (-0.47, -0.05) | -0.03 (-0.24, 0.18) | 0.12 (-0.07, 0.31) |
| **Change in BMI^d^** |  |  |  |  |  |
| Participants with obesity | 0.39 (0.14, 0.64) | -0.84 (-1.28, -0.41) | -0.79 (-1.24, -0.35) | 0.72 (0.42, 1.01) | 0.84 (0.54, 1.14) |
| Participants with overweight | 0.31 (0.23, 0.38) | -0.18 (-0.28, -0.08) | -0.22 (-0.32, -0.13) | 0.33 (0.24, 0.42) | 0.31 (0.23, 0.39) |
| Participants with normal weight | -0.17 (-0.22, -0.12) | 0.01 (-0.06, 0.07) | -0.16 (-0.22, -0.1) | 0.1 (0.04, 0.16) | 0.27 (0.22, 0.33) |
| Participants with underweight | -1.49 (-1.73, -1.24) | -0.51 (-0.98, -0.03) | -0.49 (-0.92, -0.05) | 0.48 (0.16, 0.81) | 0.5 (0.21, 0.8) |

^a^ Elevated BP was defined as either systolic BP ≥ 140 mmHg or diastolic BP ≥ 90 mmHg; ^b^ Elevated BG was defined as a BG of 10 mmol/L or higher; ^c^ Obesity was defined as body fat rate ≥ 30% for males and ≥ 42% for females; Overweight was defined as body fat rate ≥ 25% for males and ≥ 36% for females. ^d^Obesity was defined as BMI ≥ 30 kg/m^2^; Overweight was defined as 30 kg/m^2^ ≥ BMI > 25 kg/m^2^; Normal weight was defined as 25 kg/m^2^ ≥ BMI > 18.5 kg/m^2^. ^e^Time was transformed with a negative exponent to account for the non-linear time effect, calculated as exp(-time since start in weeks). A positive coefficient represents a decreasing trend. ^f^parenthetical values represent 95% confidence intervals

Table S11. Mediating analysis of program engagement on BP, and BG through the change in body fat percentage.

| Healh indicators | Indirect effect | |  | Direct effect | |  | Total effect | |
| --- | --- | --- | --- | --- | --- | --- | --- | --- |
|  | Medium vs. Low | High vs. Low |  | Medium vs. Low | High vs. Low |  | Medium vs. Low | High vs. Low |
| **Change in Systolic Blood Pressure** |  |  |  |  |  |  |  |  |
| Participants with elevated BP | **0.004 (0.001, 0.008)** | **0.011 (0.008, 0.015)** |  | **-0.342 (-0.424, -0.276)** | **-0.717 (-0.801, -0.647)** |  | **-0.338 (-0.421, -0.274)** | **-0.706 (-0.791, -0.633)** |
| Participants with normal BP | **0.007 (0.004, 0.01)** | **0.015 (0.011, 0.018)** |  | 0.026 (-0.005, 0.057) | -0.022 (-0.047, 0.005) |  | 0.033 (0.003, 0.063) | -0.007 (-0.033, 0.021) |
| Participants with obesity | -0.011 (-0.02, 0) | **-0.038 (-0.049, -0.03)** |  | -0.063 (-0.143, 0.042) | -0.073 (-0.167, 0.029) |  | -0.073 (-0.157, 0.026) | **-0.112 (-0.204, -0.018)** |
| Participants with overweight | -0.001 (-0.004, 0.003) | **-0.015 (-0.019, -0.011)** |  | -0.024 (-0.063, 0.017) | **-0.115 (-0.173, -0.055)** |  | -0.024 (-0.065, 0.017) | **-0.13 (-0.187, -0.07)** |
| Participants with healthy weight | **0.013 (0.01, 0.017)** | **0.025 (0.022, 0.028)** |  | -0.037 (-0.078, 0.012) | **-0.11 (-0.153, -0.07)** |  | -0.024 (-0.067, 0.024) | **-0.085 (-0.13, -0.046)** |
| **Change in Diastolic Blood Pressure** |  |  |  |  |  |  |  |  |
| Participants with elevated BP | **0.003 (0.001, 0.005)** | **0.007 (0.004, 0.011)** |  | **-0.186 (-0.253, -0.111)** | **-0.357 (-0.432, -0.297)** |  | **-0.183 (-0.249, -0.108)** | **-0.35 (-0.426, -0.289)** |
| Participants with normal BP | **0.004 (0.002, 0.006)** | **0.007 (0.005, 0.01)** |  | -0.008 (-0.037, 0.028) | **-0.04 (-0.077, -0.003)** |  | -0.004 (-0.033, 0.03) | -0.033 (-0.069, 0.004) |
| Participants with obesity | **-0.004 (-0.01, 0)** | **-0.013 (-0.019, -0.008)** |  | -0.086 (-0.183, 0.013) | -0.076 (-0.164, 0.016) |  | -0.09 (-0.187, 0.008) | -0.089 (-0.173, 0.003) |
| Participants with overweight | -0.001 (-0.003, 0.001) | **-0.008 (-0.011, -0.006)** |  | -0.009 (-0.056, 0.042) | **-0.087 (-0.132, -0.044)** |  | -0.01 (-0.056, 0.041) | **-0.095 (-0.139, -0.051)** |
| Participants with healthy weight | **0.006 (0.004, 0.008)** | **0.013 (0.01, 0.015)** |  | **-0.054 (-0.096, -0.012)** | **-0.092 (-0.134, -0.049)** |  | **-0.047 (-0.09, -0.005)** | **-0.079 (-0.122, -0.035)** |
| **Change in Blood Glucose** |  |  |  |  |  |  |  |  |
| Participants with elevated BG | 0.003 (-0.004, 0.014) | 0.008 (-0.005, 0.023) |  | **-0.32 (-0.57, -0.114)** | **-1.059 (-1.309, -0.822)** |  | **-0.317 (-0.566, -0.113)** | **-1.052 (-1.293, -0.817)** |
| Participants with normal BG | **0.003 (0.001, 0.005)** | **0.005 (0.003, 0.008)** |  | 0.031 (-0.024, 0.085) | 0.015 (-0.051, 0.061) |  | 0.034 (-0.021, 0.088) | 0.02 (-0.045, 0.067) |
| Participants with obesity | -0.003 (-0.014, 0.005) | **-0.019 (-0.041, -0.004)** |  | 0.029 (-0.118, 0.166) | -0.047 (-0.202, 0.116) |  | 0.026 (-0.131, 0.165) | -0.066 (-0.221, 0.099) |
| Participants with overweight | 0.002 (-0.001, 0.006) | **-0.006 (-0.01, -0.004)** |  | -0.014 (-0.113, 0.062) | **-0.124 (-0.204, -0.046)** |  | -0.012 (-0.109, 0.065) | **-0.13 (-0.211, -0.05)** |
| Participants with healthy weight | 0.002 (0, 0.004) | 0.004 (-0.001, 0.008) |  | 0.007 (-0.074, 0.074) | -0.047 (-0.125, 0.037) |  | 0.009 (-0.071, 0.075) | -0.043 (-0.119, 0.041) |

Elevated BP was defined as either systolic BP ≥ 140 mmHg or diastolic BP ≥ 90 mmHg; Elevated BG was defined as a BG of 10 mmol/L or higher; Obesity was defined as body fat rate ≥ 30% for males and ≥ 42% for females; Overweight was defined as body fat rate ≥ 25% for males and ≥ 36% for females. Age, gender, BMI, marital status, education, region, season, presence of diabetes, and presence of hypertension were adjusted as covariates. Bolded variables indicate statistical significance (*p* ≤ 0.05)


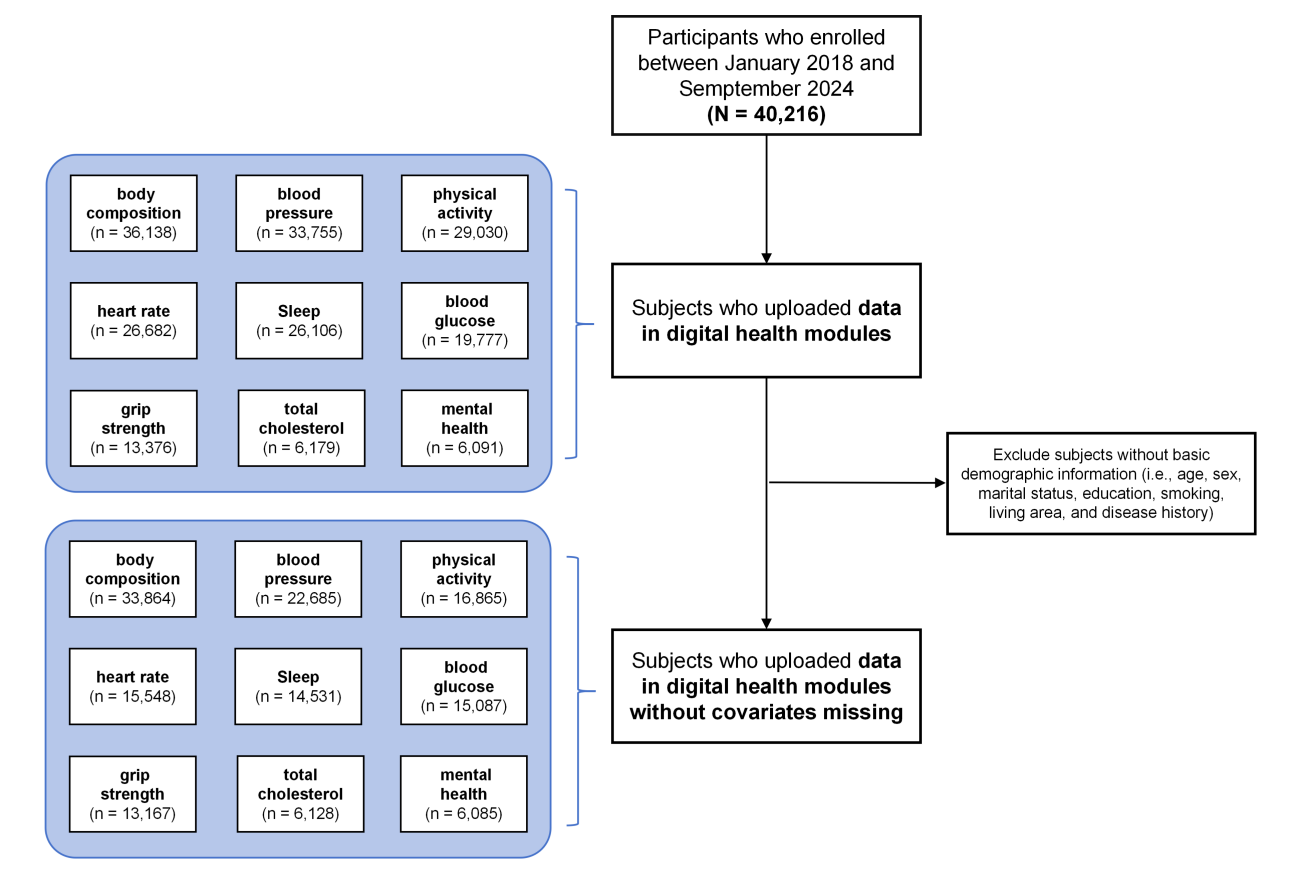


**Figure S1.** Flow chart of the study design.

Note: For heart rate data we additionally excluded those without recordings from valid days (wear time > 5h)


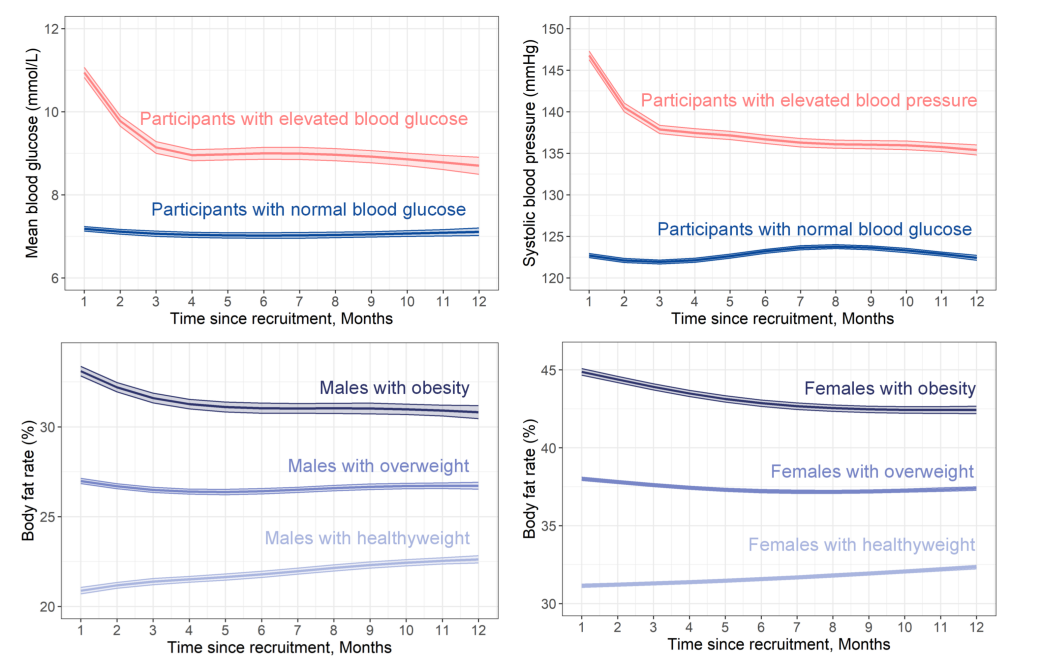


**Figure S2.** BG, BP, and body fat percentage change over 12-month follow-up with each month having at least four measurements. BG, blood glucose; BP, blood pressure. Elevated BG was defined as a BG of 10 mmol/L or higher; Elevated BP was defined as either systolic BP ≥ 140 mmHg or diastolic BP ≥ 90 mmHg; Obesity was defined as body fat rate ≥ 30% for males and ≥ 42% for females; Overweight was defined as body fat rate ≥ 25% and ≥ 36%.
